# App-Based Mindfulness for Attenuation of Subjective and Physiological Stress Reactivity in a Population With Elevated Stress: Randomized Controlled Trial

**DOI:** 10.2196/47371

**Published:** 2023-10-13

**Authors:** Ulrich Kirk, Walter Staiano, Emily Hu, Christelle Ngnoumen, Sarah Kunkle, Emily Shih, Alicia Clausel, Clare Purvis, Lauren Lee

**Affiliations:** 1 Department of Psychology University of Southern Denmark Odense Denmark; 2 Department of Physical Education and Sport University of Valencia Valencia Spain; 3 Headspace Inc Santa Monice, CA United States; 4 Department of Psychology Harvard University Cambridge, MA United States; 5 Clinical Excellence Research Center School of Medicine Stanford University Stanford, CA United States

**Keywords:** Mindfulness, mental health, stress, smartphone, technology, Headspace, mobile phone

## Abstract

**Background:**

Stress-related mental health disorders have steadily increased and contributed to a worldwide disease burden with up to 50% experiencing a stress-related mental health disorder worldwide. Data suggest that only approximately 20%-65% of individuals receive treatment. This gap in receiving treatment may be attributed to barriers such as limited treatment access, negative stigma surrounding mental health treatment, approachability (ie, not having a usual treatment plan or provider), affordability (ie, lack of insurance coverage and high treatment cost), and availability (ie, long waits for appointments) leaving those who need treatment without necessary care. To mitigate the limited access mental health treatment, there has been a rise in the application and study of digital mental health interventions. As such, there is an urgent need and opportunity for effective digital mental health interventions to alleviate stress symptoms, potentially reducing adverse outcomes of stress-related disorders.

**Objective:**

This study examined if app-based guided mindfulness could improve subjective levels of stress and influence physiological markers of stress reactivity in a population with elevated symptoms of stress.

**Methods:**

The study included 163 participants who had moderate to high perceived stress as assessed by the Perceived Stress Scale (PSS-10). Participants were randomly allocated to 1 of 5 groups: a digital guided program designed to alleviate stress (Managing Stress), a digital mindfulness fundamentals course (Basics), digitally delivered breathing exercises, an active control intervention (Audiobook), and a Waitlist Control group. The 3 formats of mindfulness interventions (Managing Stress, Basics, and Breathing) all had a total duration of 300 minutes spanning 20-30 days. Primary outcome measures were perceived stress using the PSS-10, self-reported sleep quality using the Pittsburgh Sleep Quality Index, and trait mindfulness using the Mindful Attention Awareness Scale. To probe the effects of physiological stress, an acute stress manipulation task was included, specifically the cold pressor task (CPT). Heart rate variability was collected before, during, and after exposure to the CPT and used as a measure of physiological stress.

**Results:**

The results showed that PSS-10 and Pittsburgh Sleep Quality Index scores for the Managing Stress (all *P*<.001) and Basics (all *P*≤.002) groups were significantly reduced between preintervention and postintervention periods, while no significant differences were reported for the other groups. No significant differences among groups were reported for Mindful Attention Awareness Scale (*P*=.13). The physiological results revealed that the Managing Stress (*P*<.001) and Basics (*P*=.01) groups displayed reduced physiological stress reactivity between the preintervention and postintervention periods on the CPT. There were no significant differences reported for the other groups.

**Conclusions:**

These results demonstrate efficacy of app-based mindfulness in a population with moderate to high stress on improving self-reported stress, sleep quality, and physiological measures of stress during an acute stress manipulation task.

**Trial Registration:**

ClinicalTrials.gov NCT05832632; https://www.clinicaltrials.gov/ct2/show/NCT05832632

## Introduction

### Background

Elevated stress is a state in which an individual experiences excessive or prolonged psychological and physiological strain [1,2]. The impact of elevated stress is wide-ranging and can affect various aspects of an individual's life. Sustained and elevated stress has been shown to be associated with adverse health effects such as obesity, type 2 diabetes, and cardiovascular disease [1-3]. Stress-related disorders are also a contributing factor to the onset of a range of mental health disorders, including depression and anxiety [4-9] and a heightened risk of cardiovascular disease [10]. As such, the connection between stress-related disorders, mental health disorders, and negative physical outcomes is well-established. Therefore, it is important to develop evidence-based mental health interventions to help reduce stress and therefore mitigate the adverse outcomes of stress-related disorders.

Mindfulness is defined as a state of being attentive to and bringing awareness to sensations that are taking place in the present moment without judgment [11]. Mindfulness is considered an evidence-based practice where one aims to reduce the effects of stress both psychologically and physiologically [11,12]. The most well-known mindfulness-based intervention is mindfulness-based stress reduction (MBSR) [11], which is delivered in group settings where participants meet on 8 weekly sessions and an experienced mindfulness teacher guides the sessions, provides instructions, and facilitates discussions. MBSR has been shown to be effective for managing stress and its detrimental effects on mental and physical health [13]. Research has shown that MBSR can lead to improved psychological well-being, reduced anxiety, and depressive symptoms, and enhanced overall resilience to stress [14-16].

However, the MBSR program is time consuming and costly. In recent years digital mental health interventions have been developed using self-guided mindfulness-based interventions [17,18]. Specifically, several research studies suggest that the Headspace mindfulness app decreases subjective levels of stress [19-26] and increases sleep quality [27]. Despite growing demands and usage of digital mental health apps, there is a lack of evidence on the efficacy of these interventions in clinical populations [28,29]. That is, research has primarily focused on effects of usage of digital mindfulness apps and interventions in healthy populations [20-26] and found reduced levels of stress in college students, nurses and in the workplace. However, there are a few studies that have looked at the effects of digital mindfulness apps in clinical populations [30,31]. Specifically, a meta-analysis included 15 randomized controlled trials to measure the effect of digital mindfulness interventions and reported significant effects on depression, anxiety, and stress [32]. Interestingly, the study reported higher effect sizes for digital mindfulness interventions with therapist guidance than for digital mindfulness without therapist guidance. Another study using a digital mindfulness intervention found reductions in psychiatric symptoms in adolescents undergoing mental health treatment [33].

Research has also found that mindfulness may improve cognitive functioning in healthy participants, which refers to the mental activities involved in maintaining and acquiring and using information. Specifically, studies using the Headspace mindfulness app have shown that 4 weeks of app-based mindfulness practice reduces behavioral indicators of mind wandering [19,34]. Mind wandering refers to the phenomenon in which the mind drifts away from the current task or focus of attention and becomes engaged in spontaneous thoughts unrelated to the present moment. Separate lines of research have discussed the component of *attention monitoring* embedded in mindfulness as a mechanism that may explain how mindfulness improves cognitive functioning outcomes by reducing mind wandering [35]. An additional component of mindfulness is *acceptance*, which according to the authors is necessary for reducing affective reactivity, such that attention monitoring and acceptance skills together act to improve negative affect and stress-related health outcomes and may explain the mechanism of action behind why mindfulness is thought to influence stress.

### Effects of Mindfulness on Physiological Stress Reactivity

In addition to its psychological and cognitive effects, mindfulness has been found to have physiological effects that reduce stress. Regular mindfulness practice has been associated with decreased blood pressure, increased heart rate variability (HRV), and reduced cortisol levels [36-39], which together demonstrate the underlying physiological mechanism of why mindfulness is thought to influence stress-related health outcomes.

Elevated stress is widely regarded as healthy and functional when confronted with acute stressful situations, however permanent exposure to elevated stress—and in particular the failure to recover from stress—may lead to dysfunction of the underlying neurobiology [40]. The sympathetic nervous system (SNS) is one of the 2 main divisions of the autonomic nervous system (ANS), the other being the parasympathetic nervous system (PNS). The SNS plays a crucial role in mobilizing the body's response to perceived threats or stressful situations [41]. The PNS operates in opposition to the SNS and is responsible for facilitating bodily processes during periods of rest. The SNS increases the heart’s cardiac output and decreases HRV, which is needed during acute stressful situations. Conversely, the PNS slows the heart rate and increases HRV to restore homeostasis. This natural interplay between these 2 systems allows the heart to quickly respond to different situations and needs based on the context [42]. It is generally assumed that HRV, a measure of beat-to-beat variability in heart rate, is mediated by the ANS [43,44]. Currently, there is not a universally recognized standard for stress evaluation [45]. However, studies on HRV and stress reactivity are increasing in frequency [45]. Evidence suggests that the physiological measure of HRV is impacted by stress and supports its use when assessing psychological health and stress [45]. Thus, we used HRV in this study to measure the effect of how digital mindfulness interventions impact physiological markers of stress.

Research studies have found that engaging in mindfulness can increase HRV [36-39,46,47]. Taken together, these studies suggest that mindfulness activates the PNS and promotes a state of relaxation. A higher HRV indicates a flexible ANS that can adapt to changing circumstances and shift between states of activation (SNS) and relaxation (PNS). By increasing HRV, mindfulness may thus improve emotional regulation, reduce stress reactivity, and enhance resilience to stress.

### Effects of Mindfulness on Acute Stress

The cold pressor task (CPT) is a laboratory test commonly used to induce a physiological stress response in participants. The CPT involves immersing the participant's hand in an ice-cold water bath for 3 minutes, which causes vasoconstriction in the submerged hand. This triggers a physiological response, which activates the SNS, leading to an increase in heart rate, blood pressure, and peripheral vasoconstriction [48-54]. The CPT is widely used as a stress manipulation that elicits and models the effects of mild to moderate acute stress that participants might encounter in their everyday life [48,49,51,53,55,56]. Studies suggest that administration of the CPT disrupts executive functioning including working memory capacity [57]. Therefore, acute stress has a profound negative impact on cognitive functioning, and thus the CPT serves as an excellent probe for interventions such as digital mindfulness to test if digital mindfulness interventions influence physiological markers of stress reactivity during exposure to acute stress. A recent study demonstrated that administration of the Headspace mindfulness app for a period of 4 weeks mediated the relationship between cognitive performance and acute stress [24]. The results showed that the digital mindfulness intervention uncoupled the relationship between cognitive performance and acute stress, meaning that participants who underwent mindfulness training were less affected by stress during cognitive performance compared to the control group. The findings of this study suggest that mindfulness training may be a useful approach to mitigate the negative impact of acute stress.

### Aims of the Study

This study examined if 3 different formats of digital mindfulness interventions demonstrated efficacy in terms of reducing self-reported levels of stress, sleep quality, and influencing physiological markers of stress reactivity in a population with elevated levels of stress.

To accomplish the experimental aim, participants with moderate and high stress according to the Perceived Stress Scale (PSS-10) [58] were randomized to 1 of 5 groups (3 formats of mindfulness, 1 active control, and 1 Waitlist Control). The study investigated if changes in subjective stress (PSS-10), sleep quality (PSQI) and trait mindfulness (Mindful Attention Awareness Scale [MAAS]) showed differences between the preintervention and postintervention periods.

The 3 types of mindfulness interventions were identical in total training dosage but varied in content and intervention length. That is, 2 of the 3 mindfulness interventions (Basics and Breathing) consisted of 30 sessions, whereas the third intervention (Managing Stress) consisted of 20 sessions. The type of content also varied whereby 2 interventions (Basics and Managing Stress) were programmatic that progressed from session to session, whereas the third interventions (Breathing) consisted of single succinct exercises. Furthermore, 1 intervention was designed specifically to reduce stress in people with elevated stress using mindfulness-based content (Managing Stress). This interventional setup allowed us to investigate if the unique characteristic of each intervention would result in differential efficacy in a population with moderate to high stress, while keeping the total training duration of each intervention identical. In other words, the rationale for employing 3 formats of mindfulness interventions was to explore if there were any differential effects between these distinct formats of mindfulness.

To probe psychological (self-reported) effects of stress, the study examined if changes in stress (PSS-10), sleep quality (PSQI), and mindfulness (MAAS) showed differences between the preintervention and postintervention periods.

We hypothesized (H1) that the app-based mindfulness interventions would yield significant improvements in self-reported stress (PSS-10) as compared to the active and Waitlist Control groups. We also hypothesized that trait mindfulness (MAAS) and sleep quality (PSQI) would improve in the mindfulness groups compared to control groups.

To probe physiological effects of stress, the study employed an acute stress manipulation task (ie, the CPT) at the preintervention and postintervention stage while measuring physiological activity (HRV) before, during, and after exposure to the acute stressor, and in addition self-reported stress perception immediately after stress exposure.

We hypothesized (H2) that the app-based mindfulness interventions would result in reduced physiological stress reactivity during exposure to the CPT, expressed as increased HRV activity compared to the active and Waitlist Control groups.

## Methods

### Participants

**Figure 1:**
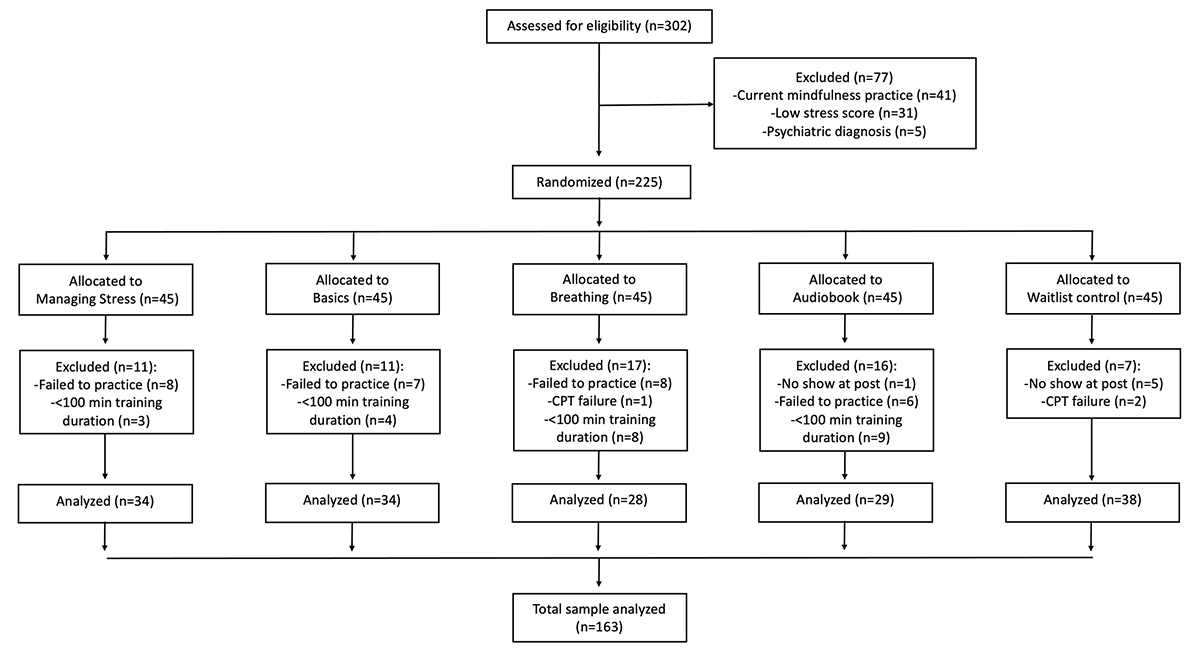
CONSORT flow diagram showing the number of participants in each group and the phases of the study from enrollment to analysis. CPT: cold pressor task; CONSORT: consolidated standards of reporting trials.

A total of 225 research participants who had moderate to high perceived stress based on PSS-10 total scores (14-40) were recruited for the study (see Figure 1). Additional inclusion criteria were men and women between 18 and 60 years of age. Exclusion criteria were any medical diagnosis, for example, psychiatric or neurological conditions. The reason for these exclusion criteria was to limit comorbidity in our sample by recruiting participants with elevated PSS-10 scores. Other exclusion criteria were regular mindfulness practice for more than 1 month within the last year.

In total, 38 participants were excluded from the analysis either because they did not initiate the intervention practice (n=29), did not show up for the postintervention laboratory visit (n=6) or were unable to complete the CPT procedure (n=3). 24 participants were excluded because they did not meet the minimum engagement dosage of 100 minutes (total engagement duration was 300 minutes). Thus, the total number of participants in our analysis was 163 participants. The rationale for the cut off at a minimum engagement dosage of 100 minutes was to ensure that there were no significant differences in engagement duration across the 4 active intervention groups (Table 1).

**Table 1:** Demographic data for the 5 groups, shown as mean and SD collected before and after the intervention.

		Managing Stress	Basics	Breathing	Audiobook	Waitlist Control
Number (females), mean (SD)		34 (17)	34 (18)	28 (13)	29 (14)	38 (18)
Age (years), mean (SD)		24.9 (2.2)	25.3 (2.1)	25.3 (2.3)	25.9 (2.0)	25.7 (2.3)
**Race** **or** **ethnicity, n (%)**						
	White	23 (67.6)	21 (61.7)	16 (57.1)	19 (65.5)	25 (65.7)
	Hispanic	0 (0)	1 (2.9)	2 (7.1)	1 (3.4)	2 (5.2)
	Asian	5 (14.7)	4 (11.7)	4 (14.2)	4 (13.7)	4 (10.5)
	Black	3 (8.8)	3 (8.8)	3 (10.7)	2 (6.8)	2 (5.2)
	Middle Eastern	3 (8.8)	5 (14.7)	3 (10.7)	3 (10.3)	5 (13.1)
Engagement duration (minutes)^a^, mean (SD)		187.5 (72.2)	172.3 (64.1)	163.2 (69.0)	164.8 (57.9)	N/A^b^

^a^The amount of engagement duration was calculated as the total minutes of engagement during the intervention period.

^b^N/A: not applicable.

### Ethical Considerations

The participants were recruited through flyers and advertisements at a local university (University of Southern Denmark) and the Region of Southern Denmark. All procedures were conducted in accordance with the local ethical committee (*Videnskabsetisk Komité for Region Syddanmark - project ID: S-20170199*). The study was performed in accordance with the ethical standards laid down in the 1964 Declaration of Helsinki and its later amendments. Participants received compensation for their participation in the study corresponding to a DKK 300 gift card (approximately US $45). All participants provided written consent prior to participation in the study.

### Experimental Procedures

The study included 5 experimental groups using a pre-post design.

Overall, 48 hours prior to the laboratory visit, eligible participants were emailed instructions to refrain from alcohol and nicotine before coming to the laboratory to avoid known influences of these factors on autonomic activity [59-61]. Participants were instructed not to engage in intense physical activity for the 48-hour period but were otherwise asked to maintain their daily and nightly routines. Both the pre- and postintervention laboratory visits were conducted on weekdays between 11 AM and 4 PM. This time window was kept constant across measurement periods to reduce variance of the circadian rhythm [62,63].

**Figure 2:**
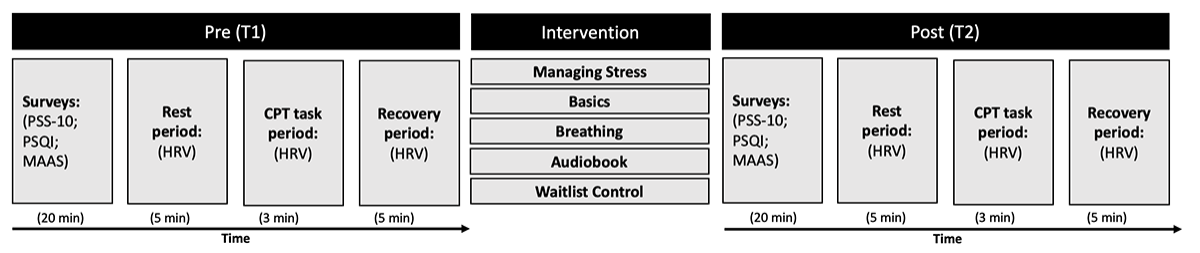
Outline of the study procedures. CPT: cold pressor task; HRV: heart rate variability; MAAS: Mindful Attention Awareness Scale; PSQI: Pittsburgh Sleep Quality Index; PSS-10: Perceived Stress Scale.

Upon arrival for the first laboratory visit (ie, preintervention or T1), the participants initially signed the consent form. Participants were subsequently instructed on how to complete the experimental procedures (Figure 2). The experimental procedures consisted of completing surveys (see *psychological measures*), and subsequently completing the CPT, that is, the stress manipulation whilst HRV was collected (see *physiological measures*). Specifically, participants were seated in a chair while the physiological HRV monitor was applied using 2 electrodes. After making sure that the HRV monitor was correctly applied, the participants were instructed about the CPT. The participants were asked to rest for 5 minutes by sitting in a chair in an upright position during the *rest period*. Subsequently, participants were instructed to initiate the CPT procedure (*CPT period*; see *acute stress manipulation*). Having completed the stress manipulation, that is, CPT, the participants were instructed to rest for 5 minutes by sitting in a chair in an upright position (*recovery period*).

The randomization procedure was determined after study recruitment, but before study launch. Specifically, participants were randomly allocated to 1 of 5 groups (Managing Stress, Basics, Breathing, Audiobook, or Waitlist Control) using sequence generation by the research team, who were not formally blinded to group allocation. Participants were not informed about group allocation until after completion of the preintervention experimental procedures. HRV was collected continuously during the CPT procedure. That is, the CPT procedure was broken up into 3 parts: *rest period, CPT period*, and *recovery period* (Figure 2). Following the preintervention laboratory visit, participants were instructed to initiate the 30-day intervention starting the following day. The second laboratory visit procedures (ie, postintervention or T2) were identical to the experimental procedures explained above.

### Psychological Measures and Physiological Measures

The experimental methods and outcome measures are described in detail in the Multimedia Appendix 1 [64-68].

### Interventions

The interventions were completed using a custom-built research smartphone app. Participants were given access to a smartphone app that was built for the purpose of conducting scientific research at the University of Southern Denmark that contained the training content for each specific intervention. The app has been applied in previous scientific research studies [27,36,69]. The app was set up to contain specific content related to each of the interventions. That is, participants could only access the content in the app belonging to the specific type of intervention they were completing at any given time. Compliance was provided by a backend in the app that tracked the timestamps to keep count of content length belonging to each intervention for each participant.

Participants did not receive an introductory session to the active intervention conditions but were provided with oral and written instructions about the app usage for the intervention period. Participants were instructed to follow the program in full.

### Managing Stress (Mindfulness) Intervention

The content of the training was provided by Headspace [70] and based on well-established concepts and practices within the stress [71-74], stress management [75,76], and mindfulness training [11] literature. The program involved a daily combination of evidence-based stress management techniques including an advice video featuring stress psychoeducation, a mindfulness meditation, a relaxation method (ie, progressive muscle relaxation), and a reflective activity (ie, gratitude prompts). All were delivered through short, animated videos and sound files in the app. The Managing Stress program has been tailored for people with elevated stress levels (Figure S2 in Multimedia Appendix 2). Whereas the other 3 active interventions comprised 30 sessions, the Managing Stress intervention entailed 20 total sessions (5 sessions per week) with an average of 15 minutes duration per session. The Managing Stress intervention had the same total duration relative to the duration of the other 3 active interventions (approximately 300 min in total for each intervention), allowing us to investigate if the training frequency yielded a difference across the active interventions.

### Basics (Mindfulness) Intervention

The content of the training was provided by Headspace [70] and based on well-established concepts and practices within the mindfulness literature [11]. The course entailed daily practice in guided mindfulness meditation, with instructions delivered through short, animated videos and sound files in the app (Figure S1 in Multimedia Appendix 3). The training course centered on mindfulness meditation, which included focusing on a selected object (ie, the body or the breath), monitoring the activity of the mind, noticing mind-wandering, and developing a nonjudgmental orientation toward one’s experience (ie, equanimity). The Basics intervention entailed daily practice sessions of 10-minute duration for a total of 30 sessions.

### Breathing (Mindfulness) Intervention

The content of the training was provided by Headspace [70] and based on well-established literature on the positive effects of deep breathing and diaphragmatic breathing on stress [77,78]. The Breathing intervention consisted of brief (around 1 min) guided deep breathing and diaphragmatic breathing exercises. There were 9 different deep breathing exercises that all contained identical instructions. The participants in this group were instructed to freely choose from the exercises and complete 10 exercises (corresponding to 10 min) per day for a total of 30 days. Importantly, this intervention was different from the 2 other mindfulness interventions in that it did not contain programmatic content that progressed over time, but rather consisted of single succinct exercises. However, it is to be noted that similar deep breathing exercises were part of all of the mindfulness interventions, including the Managing Stress, the Basics, and the Breathing interventions.

### Audiobook Intervention

Mindfulness has been hypothesized to train attention and affect through interoceptive nonjudgmental awareness [79-81]. Therefore, to deliberately manipulate the active control intervention, this study aimed to de-emphasize these elements by isolating the mechanisms of action in mindfulness. The participants in the active control intervention therefore received instructions to listen to an audiobook. Audiobooks have been used previously as active control for mindfulness in several studies [82-86]. Specifically, in accordance with previous literature we used the following audiobook in this study: “The Natural History of Selborne” by White and Taylor [87]. The general topic of the audiobook contains observations of natural history of the area around Selborne organized more or less systematically by species and group. The intervention entailed listening to the audiobook for a daily duration of 10 minutes for a total of 30 sessions.

### Waitlist Control

The Waitlist Control group required that participants did not follow an intervention. However, the Waitlist Control group was given the option to obtain access to 1 of the 3 active interventions after completion of the study.

### Statistical Analysis

All data are presented as mean ± 1 SD unless otherwise stated. Assumptions of statistical tests for normal distribution and sphericity of data were checked. A series of mixed groups (Managing Stress, Basics, Breathing, Audiobook, Waitlist control) × time (pretest, posttest) ANOVAs were performed on PSS-10, PSQI, MAAS, and CPT self-reported stress. A series of mixed groups (Managing Stress, Basics, Breathing, Audiobook, Waitlist control) × time (pre, post) × task period (rest, during task, and recovery) ANOVAs were performed on HRV. Significant 3-way interactions were followed-up by Group by Time ANOVAs at each time point, and 2-way interactions were followed-up with relevant corrected pairwise comparisons using the Bonferroni method (post hoc analysis) for simple main effects within each group. Where no significant interactions were found, main effects of time, group, and task period were reported. Significance level was set at 0.05 (2-tailed) for all analyses. The effect sizes for the ANOVAs were calculated as partial eta squared (η²p), with 0.02, 0.13, and 0.26 indicating small, medium, and large effects, respectively. Data analysis was conducted using SPSS (version 27; IBM Corp).

## Results

### Measures at Baseline (Pretest)

Age, gender, race, or ethnicity, and engagement duration (in minutes) is presented in Table 1, and behavioral measures (PSS-10, PSQI, MAAS, and self-reported stress during CPT) are presented in Table 2. No statistically significant differences were detected for any of these variables at baseline.

**Table 2:** Behavioral data for the 5 groups collected pre- and postintervention and percentage change in self-reported measures.

	Managing Stress	Basics	Breathing	Audiobook	Waitlist Control
PSS-10^a^ (Pre), mean (SD)	21.6 (3.2)	21.6 (4.7)	22.0 (4.1)	22 (1.42)	21.7 (4.4)
PSS-10 (Post), mean (SD)	18.6 (3.0)	17.4 (4.3)	22.7 (3.5)	23.6 (5.0)	21.8 (4.0)
PSS-10 percentage change, %	–13.8	–14	3.1	7.2	0.4
PSQI^b^ (Pre), mean (SD)	7.3 (1.4)	7.3 (1.3)	7.2 (1.6)	7.2 (1.5)	7.2 (1.7)
PSQI (Post), mean (SD)	6.1 (1.3)	6.4 (1.3)	7.0 (1.7)	7.1 (1.4)	7.3 (1.7)
PSQI percentage change, %	–16.4	–12.3	–2.7	–1.3	1.3
MAAS^c^ (Pre), mean (SD)	3.0 (0.9)	2.9 (1.0)	2.9 (1.1)	3.0 (1.0)	2.8 (1.2)
MAAS (Post), mean (SD)	3.7 (1.3)	3.4 (1.1)	2.8 (1.0)	2.9 (1.2)	2.9 (1.2)
MAAS percentage change, %	23.3	17.2	–3.4	–3.3	3.5
CPT^d^ self-reported stress (Pre), mean (SD)	7.5 (1.3)	7.3 (1.3)	7.9 (1.3)	7.4 (1.3)	7.7 (1.3)
CPT self-reported stress (Post), mean (SD)	6.9 (1.4)	6.9 (1.3)	7.2 (1.5)	7.2 (1.5)	7.3 (1.4)
CPT self-reported stress percentage change, %	–8	–5.4	–8.8	–2.7	–5.1

^a^PSS-10: Perceived Stress Scale.

^b^PSQI: Pittsburgh Sleep Quality Index.

^c^MAAS: Mindful Attention Awareness Scale.

^d^CPT: cold pressor task.

### Psychological Measures: PSS-10, PSQI, MAAS, and CPT Self-Reported Stress

**Figure 3:**
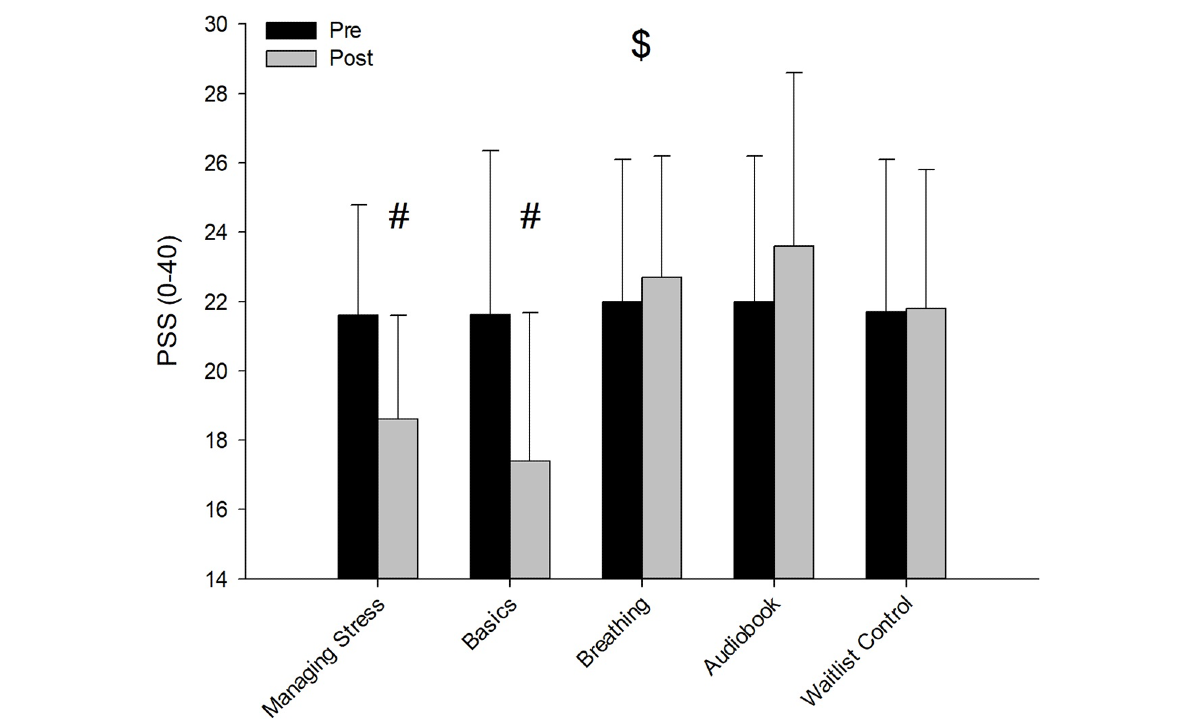
PSS-10 results pre and post across the 5 groups. $: interaction effect; #: simple main effects of time (follow-ups). PSS-10: Perceived Stress Scale.

There was an interaction for PSS-10 across the 5 groups and time (*F*_4,158_=6.964; *P*<.001; η²p=0.188). Follow-up tests revealed that the PSS-10 score for the Managing Stress (*P*<.001; η²p=0.096) and the Basics (*P*=.01; η²p=0.060) groups were significantly reduced from the preintervention to the postintervention period, while no significant differences were reported for Breathing (*P*=.51; η²p=0.003), Audiobook (*P*=.08; η²p=0.007) and Control (*P*=.75; η²p=0.001; Figure 3; Table 2).

**Figure 4:**
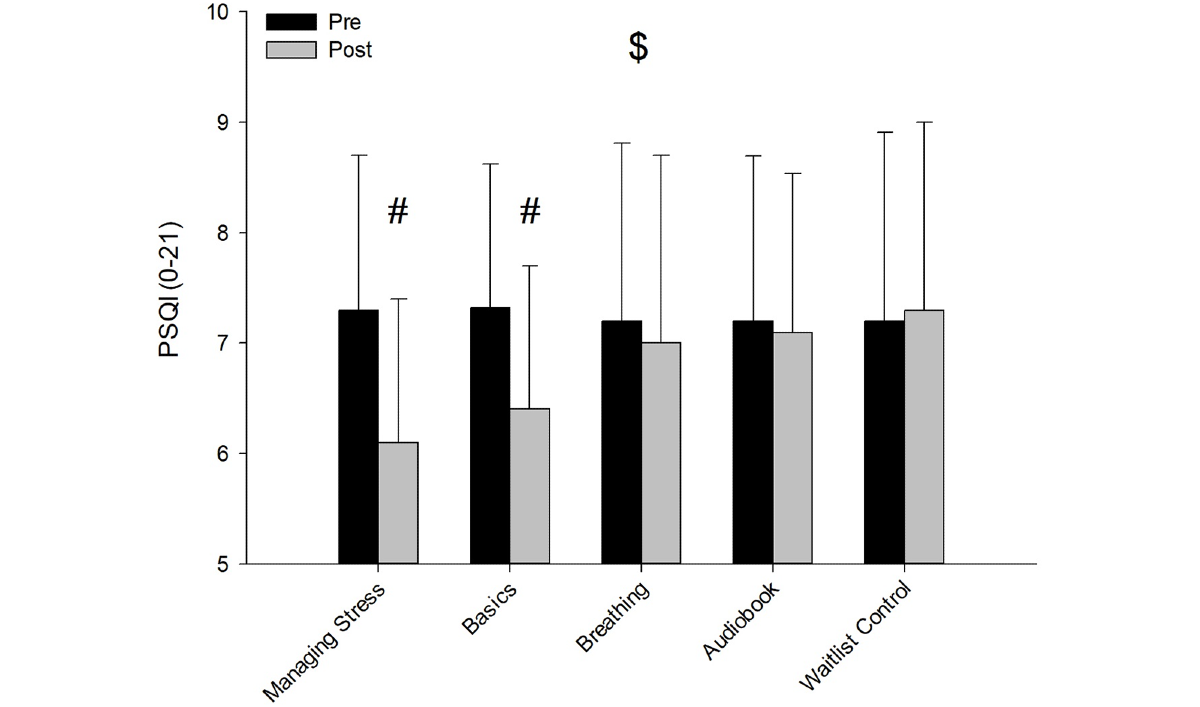
PSQI results pre (A) and post (B) across the 5 groups. $: interaction effect; #: simple main effects of time (follow-ups). PSQI: Pittsburgh Sleep Quality Index.

There was an interaction for PSQI across the 5 groups and time (*F*_4,158_=4.941; *P=*.001; η²p=0.111). Follow-up tests revealed that the PSQI score for the Managing Stress (*P*<.001; η²p=0.131) and the Basics (*P*=.001; η²p=0.074) groups were significantly reduced from the preintervention to the postintervention period, while no significant differences were reported for Breathing (*P*=.50; η²p=0.003), Audiobook (*P*=.60; η²p=0.002), and Control (*P*=.73; η²p=0.001; Figure 4; Table 2).

There was no significant interaction (*F*_4,158_=1.801; *P=*.13; η²p=0.044) or main effects of time (*F*_1,158_=1.957; *P=*.16; η²p=0.012) and group (*F*_4,158_=2.245; *P=*.07; η²p=0.054) for MAAS (Table 2).

No interaction (*F*_4,158_=0.279; *P=*.90; η²p=0.007) or main effect of group (*F*_4,158_=1.639; *P=*.17; η²p=0.040) were found for self-reported stress during the CPT (Table 2). A main effect of time was observed (*F*_1,158_=10.960; *P*=.001; η²p=0.065).

### Physiological Measures: HRV

**Figure 5:**
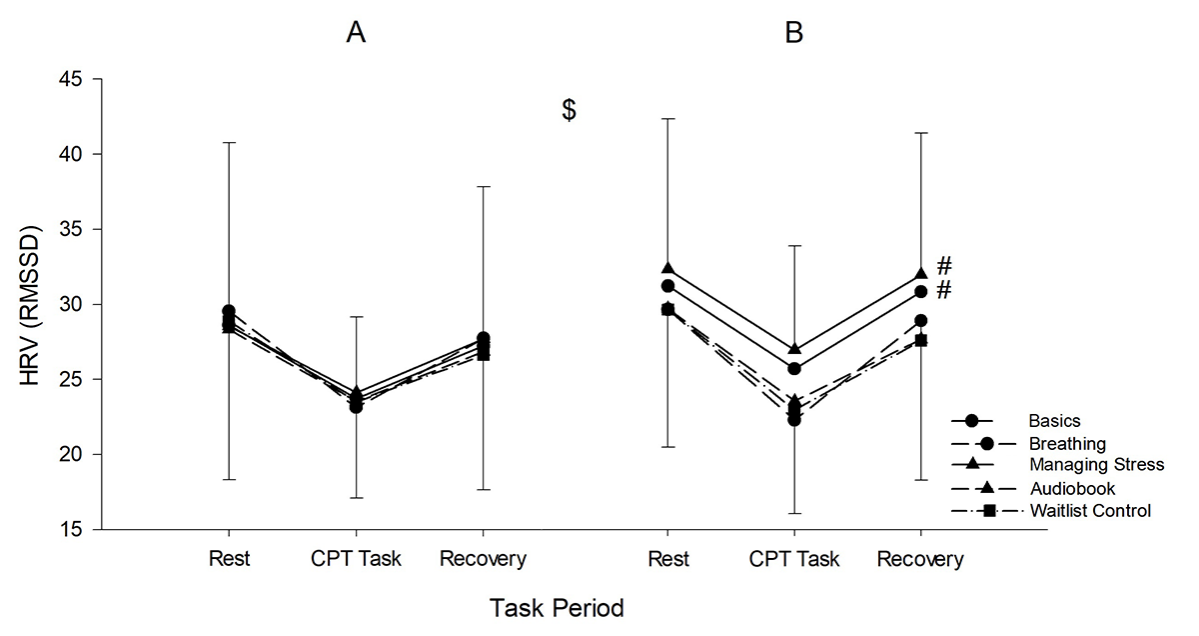
HRV results pre and post across the 5 groups. $: interaction effect; #: simple main effects of time (follow-ups). HRV: heart rate variability; RMSSD: root-mean-square of successive differences between normal heartbeats.

No 3-way interaction (group × time × task period) was detected for HRV (*F*_8,316_=0.030; *P=*.997; η²p=0.001). However, a significant 2-way interaction (group × time [*F*_4,158_=2.637; *P*=.04; η²p=0.063]) was detected. Follow-up *t* tests revealed that HRV significantly increased from pretest to posttest for the Managing Stress (*P*<.001; η²p=0.089) and the Basics (*P*=.008; η²p=0.043) while no significant differences were reported for Breathing (*P*=.92; η²p=0.001), Audiobook (*P*=.53; η²p=0.002), and Control (*P*=.62; η²p=0.002). No group × task period (*F*_8,316_=0.854; *P*=.56; η²p=0.021) and time × task period (*F*_2,316_=1.670; *P*=.19; η²p=0.010) interactions were observed. A significant main effect of task period was observed (*F*_2,316_=42.529; *P*<.001; η²p=0.212; Figure 5; Table 3).

**Table 3:** Behavioral data for the 5 groups, shown as mean and SD collected pre- and postintervention.

			Managing Stress, mean (SD)		Basics, mean (SD)		Breathing, mean (SD)		Audiobook, mean (SD)		Waitlist Control, mean (SD)
**HRV^a^ (RMSSD^b^; Pre)**											
	Rest period	28.9 (8.0)		28.5 (9.5)		30.9 (11.3)		29.0 (8.4)		28.8 (8.0)	
	CPT^c^ period	24.5 (6.0)		23.9 (6.6)		22.0 (5.1)		23.5 (5.9)		23.5 (6.4)	
	Recovery period	28.2 (9.1)		27.0 (9.6)		28.4 (10.0)		26.0 (8.3)		26.6 (9.3)	
**HRV (RMSSD; Post)**											
	Rest period	32.9 (10.0)		31.2 (8.8)		31.0 (9.0)		29.4 (7.9)		29.7 (8.9)	
	CPT period	27.5 (6.9)		25.7 (7.1)		21.1 (4.4)		23.5 (6.6)		23.0 (6.3)	
	Recovery period	32.8 (9.1)		30.3 (7.9)		29.6 (11.0)		27.6 (9.4)		27.6 (9.5)	

**^a^**HRV: heart rate variability.

**^b^**RMSSD: root-mean-square of successive differences between normal heartbeats.

**^c^**CPT: cold pressor task.

## Discussion

### Principal Findings

This study sought to investigate if 3 formats of digital mindfulness interventions would show efficacy in reducing subjective and physiological levels of stress in a population with elevated symptoms of stress. The study found that self-reported stress as measured by PSS-10 was significantly reduced in the Managing Stress and Basics groups from the preintervention to the postintervention period compared to the other groups. Furthermore, the study found significant improvement in self-reported sleep quality as measured by the PSQI in the Managing Stress and Basics groups from the preintervention to the postintervention period compared to the other groups. Trait mindfulness, as measured by MAAS, did not yield significant differences across any of the groups. Finally, the results showed that only the Basics and Managing Stress groups displayed significantly reduced levels of physiological stress (ie, expressed as increased HRV activity) during exposure to the acute stress manipulation task (ie, CPT).

To our knowledge this study is the first study to demonstrate stress-reducing effects of a digital mindfulness app in a population with elevated baseline stress levels. Stress is prevalent in modern society and is accepted as a contributing factor to the onset of a range of mental health disorders, including depression and anxiety [6,7,88]. These findings highlight the promise of the Headspace mindfulness app in reducing both psychological and physiological stress in people with elevated stress.

### Self-Reported Effects of Stress and Sleep Quality

The magnitude of change for the PSS-10 was larger in the Managing Stress group and the Basics groups compared to the Breathing and control groups, thus finding partial support for hypothesis 1 (H1). Our findings expand upon the results of previous studies that found digital mindfulness interventions improved stress among the general population [20-23,25,26]. This study showed efficacy among a sample composed of mostly university students with elevated stress. A meta-analysis showed that digital mindfulness interventions had significant effects on depression, anxiety, and stress, which was supported in this study in terms of stress reduction [32]. However, the meta-analysis also showed that digital mindfulness interventions with therapist guidance were more effective than for digital mindfulness without therapist guidance. Our study did not include therapist guidance, future studies could examine whether therapist-guided interventions lead to higher effect sizes. An implication from this study is that the Managing Stress and Basics interventions both improved stress in a population afflicted with elevated stress, with effect sizes being slightly higher for the Managing Stress relative to the Basics intervention for both physiological measures (ie, HRV) and psychological measures (ie, PSS-10 and PSQI). The results may point to the fact that the act of following a skill-building intervention centered around mindfulness is sufficient to show changes in these outcomes, which was present in both the Managing Stress and Basics groups. Both the Managing Stress and Basics interventions were effective despite the differences in frequency and duration of individual sessions (20 daily 15-minute sessions for Managing Stress and 30 daily 10-minute sessions for Basics). The results suggest that using mindfulness app-based interventions may be a practical approach to reducing stress in that it requires fewer resources (costs, mindfulness instructors, and brick-and-mortar clinics) where people can participate remotely with fewer practical constraints.

This study found that the PSQI indexing subjective sleep quality improved in the Managing Stress and Basics groups from the preintervention to the postintervention period. It is noteworthy that the participant’s subjective sleep quality on the PSQI did not show statistical differences from the preintervention to the postintervention period for the Breathing group. However, the HRV results support this finding in that there was not a significant difference in HRV for the Breathing intervention. A recent meta-analysis reported a significant positive effect of mindfulness on sleep quality based on the results of 6 randomized controlled trials on people with insomnia [89]. In this study, we found a reduction in perceived stress and in a separate analysis an increase in sleep quality, however, only in 2 of the 3 aforementioned mindfulness interventions, which might be due to the skill-building program in Managing Stress and Basics that yielded an effect on both outcome measures.

In contrast, we found no effects of the MAAS for any of the interventions. This finding is surprising in that previous findings have revealed that web- or app-based mindfulness has a significant impact on mindfulness, albeit with small effect sizes [32]. However, we note that both the Managing Stress and Basics interventions showed a trend toward significance on the MAAS.

### Physiological Effects of Stress

We did not find significant group differences in subjective levels of stress after administration of the CPT from the preintervention to the postintervention period. However, we did observe significant differences in HRV activity both in the expectation phase before the CPT, during the CPT, and in the recovery period after the CPT in both the Managing Stress group and the Basics group in support of hypothesis 2 (H2).

A recent study employed the CPT in the context of a 30-day app-based mindfulness intervention and showed that mindfulness training was less affected by acute stress on their cognitive performance compared to the control group suggesting that mindfulness may mitigate the negative impact of acute stress [24]. This previous finding may support the results from this study. Although this study cannot speak to the precise neurobiological mechanism underlying the elevated HRV activity observed in the Managing Stress and Basics groups, we can take advantage of our understanding of other systems involved in the stress response to interpret why these effects might occur. The stress response is driven by elevated noradrenaline levels during acute stress that reflects SNS activation [90,91]. Consequently, elevated noradrenaline levels during stress lead to the rapid decline in cognitive processing and impairment of the prefrontal cortex [92]. However, our finding of increased HRV activity in the Managing Stress and Basics group, reflects increased PNS activity [44] during acute stress which is in line with the abovementioned study showing that mindfulness in the context of the CPT does not impair cognitive functioning [24]. The results demonstrate that the Managing Stress and Basics mindfulness interventions show a stress-buffering effect compared to the other interventions, specifically by increasing PNS activity in a population afflicted with elevated levels of self-reported baseline stress. Furthermore, the results show that mindfulness can momentarily decrease stress and stress-related autonomic activity, which has also been observed in related studies, albeit in healthy populations and using salivary cortisol [93,94].

The results in this study found differential efficacy whereby only 2 of the 3 mindfulness groups, that is the Managing Stress and Basics group, but not the Breathing group, showed physiological and self-reported stress-buffering effects. This could be due to the relationship between the different ingredients of the mindfulness content across the different interventions. The content of the Breathing intervention was to engage in deep breathing and diaphragmatic breathing exercises for 10 min daily, which does not seem to translate into behavioral changes that could influence physiological reactivity as opposed to the Managing Stress and Basics interventions. This finding of differential effects between the 3 types of mindfulness is interesting. Reasons for such a difference might be found in the literature demonstrating mixed results in terms of the effect of deep breathing and diaphragmatic breathing interventions across various outcome measures. Further, 1 study found that breathing exercises did not yield pain reduction in a clinical population [95]. However, another study found effects in terms of reduced distress in the context of brief deep breathing and diaphragmatic breathing exercises, albeit this study only showed a limited immediate effect of a deep breathing exercise [96]. Thus, additional studies are required to tease apart the impact of deep breathing and diaphragmatic breathing exercises on stress outcomes and respiratory-physiological effects both in the immediate phase and more chronic effects. Further, 1 interpretation of the results from this study may be that essential components of the mindfulness interventions that were not present in the breathing exercises, such as *attention monitoring* and *acceptance* components, may be responsible for the nonsignificant finding in the breathing exercise condition. This may explain the mechanism of action behind why mindfulness is thought to influence stress in this study.

### Strengths and Limitations

Although the results of this study are promising regarding the efficacy in 2 of the 3 app-based mindfulness interventions, several limitations must be noted. First, the sample primarily comprised young university students in their twenties, thus the generalizability of our findings may be limited. Second, this study did not investigate the efficacy of the app-based interventions beyond 30 days, nor whether any of the findings were maintained (regardless of app usage) beyond this period. Future studies should consider including follow-up measurements to evaluate sustained outcomes. Third, the study did not collect endocrine measures such as cortisol to assess the acute stress response during the CPT, although the HRV-data supported, it would have been interesting to also inspect correlations between endocrine measures and HRV to be completely certain that the CPT elicited reliable stress reactivity in participants. Fourth, participants were instructed to follow the program in full, however training adherence was not checked during the intervention, and participants were not reminded by the researchers to complete the daily training in the intervention period, which might have increased adherence.

Notably, a strength of this study was its relatively diverse population in terms of race, whereby 5 different ethnicities were represented in the study. In future interventions, it is important to explore the efficacy of diverse populations both in terms of race or ethnicity, age, socioeconomic status, and education level in order to generalize the findings to a broader population. A second strength of this study is that we employed multiple mindfulness interventions and were thus able to investigate if the unique characteristic of each intervention would result in differential efficacy. A third strength of this study was that both subjective and objective measures were employed which increased the inferences made in terms of reductions in both self-reported stress and objective measures of stress in the Managing Stress and Basics interventions.

### Conclusions

In summary, our findings extend previous studies suggesting the efficacy of Headspace’s app-based mindfulness interventions, specifically the Managing Stress and Basics content, to reduce stress in populations with elevated stress levels. Specifically, we found stress-buffering effects in a relatively diverse sample of participants afflicted with elevated stress. More research in this area is needed to establish efficacy and explore the degree to which effects are sustained in the long-term. The findings presented here provide important data that may be applied to the design of future studies or mental health interventions in people who experience elevated levels of stress.

## Appendix

Multimedia Appendix 1

Multimedia Appendix 2 Managing Stress (mindfulness) guided program.

Multimedia Appendix 3 Basics (mindfulness) course.

Multimedia Appendix 4 CONSORT eHealth Checklist (V 1.6.2).

## Abbreviations

ANS: autonomic nervous system
CPT: cold pressor task
HRV: heart rate variability
MAAS: Mindful Attention Awareness Scale
MBSR: mindfulness-based stress reduction
PNS: parasympathetic nervous system
PSQI: Pittsburgh Sleep Quality Index
PSS-10: Perceived Stress Scale
SNS: sympathetic nervous system

## Data Availability

The data sets generated or analyzed during the study are available from the corresponding author upon request.

## References

[1] Ng DM, Jeffery RW (2003). Relationships between perceived stress and health behaviors in a sample of working adults. Health Psychol.

[2] Steptoe A, Kivimäki M (2012). Stress and cardiovascular disease. Nat Rev.

[3] Mikolajczyk RT, El Ansari W, Maxwell AE (2009). Food consumption frequency and perceived stress and depressive symptoms among students in three European countries. Nutr J.

[4] Cohen S, Janicki-Deverts D (2012). Who's stressed? Distributions of psychological stress in the United States in probability samples from 1983, 2006, and 2009. J Appl Soc Psychol.

[5] Tian F, Shen Q, Hu Y, Ye W, Valdimarsdóttir UA, Song H, Fang F (2022). Association of stress-related disorders with subsequent risk of all-cause and cause-specific mortality: a population-based and sibling-controlled cohort study. Lancet Reg Health Eur.

[6] Iwata M, Ota KT, Duman RS (2013). The inflammasome: pathways linking psychological stress, depression, and systemic illnesses. Brain Behav Immun.

[7] Masi G, Brovedani P (2011). The hippocampus, neurotrophic factors and depression: possible implications for the pharmacotherapy of depression. CNS Drugs.

[8] Nochaiwong S, Ruengorn C, Thavorn K, Hutton B, Awiphan R, Phosuya C, Ruanta Y, Wongpakaran N, Wongpakaran T (2021). Global prevalence of mental health issues among the general population during the coronavirus disease-2019 pandemic: a systematic review and meta-analysis. Sci Rep.

[9] Coombs NC, Meriwether WE, Caringi J, Newcomer SR (2021). Barriers to healthcare access among U.S. adults with mental health challenges: a population-based study. SSM Popul Health.

[10] Richardson S, Shaffer JA, Falzon L, Krupka D, Davidson KW, Edmondson D (2012). Meta-analysis of perceived stress and its association with incident coronary heart disease. Am J Cardiol.

[11] Kabat-Zinn J (1990). Full Catastrophe Living: Using the Wisdom of Your Body and Mind to Face Stress, Pain, and Illness.

[12] Strauss C, Cavanagh K, Oliver A, Pettman D (2014). Mindfulness-based interventions for people diagnosed with a current episode of an anxiety or depressive disorder: a meta-analysis of randomised controlled trials. PLoS One.

[13] Rosenzweig S, Reibel DK, Greeson JM, Brainard GC, Hojat M (2003). Mindfulness-based stress reduction lowers psychological distress in medical students. Teach Learn Med.

[14] Chiesa A, Serretti A (2009). Mindfulness-based stress reduction for stress management in healthy people: a review and meta-analysis. J Altern Complement Med.

[15] Jain S, Shapiro SL, Swanick S, Roesch SC, Mills PJ, Bell I, Schwartz GER (2007). A randomized controlled trial of mindfulness meditation versus relaxation training: effects on distress, positive states of mind, rumination, and distraction. Ann Behav Med.

[16] Baer RA (2009). Self-focused attention and mechanisms of change in mindfulness-based treatment. Cogn Behav Ther.

[17] Sorkin DH, Janio EA, Eikey EV, Schneider M, Davis K, Schueller SM, Stadnick NA, Zheng K, Neary M, Safani D, Mukamel DB (2021). Rise in use of digital mental health tools and technologies in the United States during the COVID-19 pandemic: survey study. J Med Internet Res.

[18] Philippe TJ, Sikder N, Jackson A, Koblanski ME, Liow E, Pilarinos A, Vasarhelyi K (2022). Digital health interventions for delivery of mental health care: systematic and comprehensive meta-review. JMIR Ment Health.

[19] Bennike IH, Wieghorst A, Kirk U (2017). Online-based mindfulness training reduces behavioral markers of mind wandering. J Cogn Enhanc.

[20] Champion L, Economides M, Chandler C (2018). The efficacy of a brief app-based mindfulness intervention on psychosocial outcomes in healthy adults: a pilot randomised controlled trial. PLoS One.

[21] Economides M, Martman J, Bell MJ, Sanderson B (2018). Improvements in stress, affect, and irritability following brief use of a mindfulness-based smartphone app: a randomized controlled trial. Mindfulness (N Y).

[22] Flett JAM, Hayne H, Riordan BC, Thompson LM, Conner TS (2019). Mobile mindfulness meditation: a randomised controlled trial of the effect of two popular apps on mental health. Mindfulness.

[23] Mascaro JS, Wehrmeyer K, Mahathre V, Darcher A (2020). A longitudinal, randomized and controlled study of app-delivered mindfulness in the workplace. J Wellness.

[24] Piil F, Axelsen JL, Staiano W, Kirk U (2021). Mindfulness passes the stress test: attenuation of behavioral markers of mind wandering during acute stress. J Cogn Enhanc.

[25] Wylde CM, Mahrer NE, Meyer RML, Gold JI (2017). Mindfulness for novice pediatric nurses: smartphone application versus traditional intervention. J Pediatr Nurs.

[26] Yang E, Schamber E, Meyer RML, Gold JI (2018). Happier healers: randomized controlled trial of mobile mindfulness for stress management. J Altern Complement Med.

[27] Kirk U, Ngnoumen C, Clausel A, Purvis CK (2022). Using actigraphy and Heart Rate Variability (HRV) to assess sleep quality and sleep arousal of three app-based interventions: sleep music, sleepcasts, and guided mindfulness. J Cogn Enhanc.

[28] Lagan S, D'Mello R, Vaidyam A, Bilden R, Torous J (2021). Assessing mental health apps marketplaces with objective metrics from 29,190 data points from 278 apps. Acta Psychiatr Scand.

[29] Schueller SM, Torous J (2020). Scaling evidence-based treatments through digital mental health. Am Psychol.

[30] Avalos LA, Aghaee S, Kurtovich E, Quesenberry C, Nkemere L, McGinnis MK, Kubo A (2020). A mobile health mindfulness intervention for women with moderate to moderately severe postpartum depressive symptoms: feasibility study. JMIR Ment Health.

[31] Ainsworth B, Stanescu S, Stuart B, Russell D, Liddiard M, Djukanovic R, Thomas M (2022). A feasibility trial of a digital mindfulness-based intervention to improve asthma-related quality of life for primary care patients with asthma. J Behav Med.

[32] Spijkerman MPJ, Pots WTM, Bohlmeijer ET (2016). Effectiveness of online mindfulness-based interventions in improving mental health: a review and meta-analysis of randomised controlled trials. Clin Psychol Rev.

[33] Hutchison M, Russell BS, Starkweather AR, Gans KM (2023). Outcomes from an online pilot mindfulness based intervention with adolescents: a comparison by categories of risk. J Child Fam Stud.

[34] Axelsen JL, Meline JSJ, Staiano W, Kirk U (2022). Mindfulness and music interventions in the workplace: assessment of sustained attention and working memory using a crowdsourcing approach. BMC Psychol.

[35] Lindsay EK, Creswell JD (2017). Mechanisms of mindfulness training: Monitor and Acceptance Theory (MAT). Clin Psychol Rev.

[36] Kirk U, Axelsen JL (2020). Heart rate variability is enhanced during mindfulness practice: a randomized controlled trial involving a 10-day online-based mindfulness intervention. PLoS One.

[37] Crosswell AD, Moreno PI, Raposa EB, Motivala SJ, Stanton AL, Ganz PA, Bower JE (2017). Effects of mindfulness training on emotional and physiologic recovery from induced negative affect. Psychoneuroendocrinology.

[38] Ditto B, Eclache M, Goldman N (2006). Short-term autonomic and cardiovascular effects of mindfulness body scan meditation. Ann Behav Med.

[39] Tang YY, Ma Y, Fan Y, Feng H, Wang J, Feng S, Lu Q, Hu B, Lin Y, Li J, Zhang Y, Wang Y, Zhou L, Fan M (2009). Central and autonomic nervous system interaction is altered by short-term meditation. Proc Natl Acad Sci U S A.

[40] Schneiderman N, Ironson G, Siegel SD (2005). Stress and health: psychological, behavioral, and biological determinants. Annu Rev Clin Psychol.

[41] Charmandari E, Tsigos C, Chrousos G (2005). Endocrinology of the stress response. Annu Rev Physiol.

[42] Thayer JF, Ahs F, Fredrikson M, Sollers JJ, Wager TD (2012). A meta-analysis of heart rate variability and neuroimaging studies: implications for heart rate variability as a marker of stress and health. Neurosci Biobehav Rev.

[43] Berntson GG, Bigger JT, Eckberg DL, Grossman P, Kaufmann PG, Malik M, Nagaraja HN, Porges SW, Saul JP, Stone PH, van der Molen MW (1997). Heart rate variability: origins, methods, and interpretive caveats. Psychophysiology.

[44] Shaffer F, McCraty R, Zerr CL (2014). A healthy heart is not a metronome: an integrative review of the heart's anatomy and heart rate variability. Front Psychol.

[45] Kim HG, Cheon EJ, Bai DS, Lee YH, Koo BH (2018). Stress and heart rate variability: a meta-analysis and review of the literature. Psychiatry Investig.

[46] Garland SN, Carlson LE, Stephens AJ, Antle MC, Samuels C, Campbell TS (2014). Mindfulness-based stress reduction compared with cognitive behavioral therapy for the treatment of insomnia comorbid with cancer: a randomized, partially blinded, noninferiority trial. J Clin Oncol.

[47] Adler-Neal AL, Waugh CE, Garland EL, Shaltout HA, Diz DI, Zeidan F (2020). The role of heart rate variability in mindfulness-based pain relief. J Pain.

[48] Lovallo W (1975). The cold pressor test and autonomic function: a review and integration. Psychophysiology.

[49] Lupien SJ, Maheu F, Tu M, Fiocco A, Schramek TE (2007). The effects of stress and stress hormones on human cognition: implications for the field of brain and cognition. Brain Cogn.

[50] Forgas JP (2011). Affective influences on self-disclosure: mood effects on the intimacy and reciprocity of disclosing personal information. J Pers Soc Psychol.

[51] Raio CM, Hartley CA, Orederu TA, Li J, Phelps EA (2017). Stress attenuates the flexible updating of aversive value. Proc Natl Acad Sci U S A.

[52] Brown CC, Raio CM, Neta M (2017). Cortisol responses enhance negative valence perception for ambiguous facial expressions. Sci Rep.

[53] Velasco M, Gómez J, Blanco M, Rodriguez I (1997). The cold pressor test: pharmacological and therapeutic aspects. Am J Ther.

[54] Victor RG, Leimbach WN, Seals DR, Wallin BG, Mark AL (1987). Effects of the cold pressor test on muscle sympathetic nerve activity in humans. Hypertension.

[55] Raio CM, Orederu TA, Palazzolo L, Shurick AA, Phelps EA (2013). Cognitive emotion regulation fails the stress test. Proc Natl Acad Sci U S A.

[56] McRae AL, Saladin ME, Brady KT, Upadhyaya H, Back SE, Timmerman MA (2006). Stress reactivity: biological and subjective responses to the cold pressor and Trier social stressors. Hum Psychopharmacol.

[57] Otto AR, Raio CM, Chiang A, Phelps EA, Daw ND (2013). Working-memory capacity protects model-based learning from stress. Proc Natl Acad Sci USA.

[58] Cohen S, Kamarck T, Mermelstein R (1983). A global measure of perceived stress. J Health Soc Behav.

[59] Heart Rate Variability (1996). Standards of measurement, physiological interpretation, and clinical use. Task force of the European society of cardiology and the North American society of pacing and electrophysiology. Eur Heart J.

[60] Sjoberg N, Saint DA (2011). A single 4 mg dose of nicotine decreases heart rate variability in healthy nonsmokers: implications for smoking cessation programs. Nicotine Tob Res.

[61] Ralevski E, Petrakis I, Altemus M (2019). Heart rate variability in alcohol use: a review. Pharmacol Biochem Behav.

[62] Weitzman ED, Fukushima D, Nogeire C, Roffwarg H, Gallagher TF, Hellman L (1971). Twenty-four hour pattern of the episodic secretion of cortisol in normal subjects. J Clin Endocrinol Metab.

[63] Krieger DT, Allen W, Rizzo F, Krieger HP (1971). Characterization of the normal temporal pattern of plasma corticosteroid levels. J Clin Endocrinol Metab.

[64] Buysse DJ, Reynolds CF, Monk TH, Berman SR, Kupfer DJ (1989). The pittsburgh sleep quality index: a new instrument for psychiatric practice and research. Psychiatry Res.

[65] Brown KW, Ryan RM (2003). The benefits of being present: mindfulness and its role in psychological well-being. J Pers Soc Psychol.

[66] Ottaviani C, Shahabi L, Tarvainen M, Cook I, Abrams M, Shapiro D (2015). Cognitive, behavioral, and autonomic correlates of mind wandering and perseverative cognition in major depression. Front Neurosci.

[67] Parak J, Tarniceriu A, Renevey P, Bertschi M, Delgado-Gonzalo R, Korhonen I (2015). Evaluation of the beat-to-beat detection accuracy of PulseOn wearable optical heart rate monitor. Annu Int Conf IEEE Eng Med Biol Soc.

[68] Tarvainen MP, Niskanen JP, Lipponen JA, Ranta-Aho PO, Karjalainen PA (2014). Kubios HRV—heart rate variability analysis software. Comput Methods Programs Biomed.

[69] Kirk U, Ngnoumen C, Clausel A, Kennedy C (2022). Effects of three genres of focus music on heart rate variability and sustained attention. J Cogn Enhanc.

[70] Make it your year with Headspace.

[71] Cohen S, Kessler RC, Gordon LU, Cohen S, Kessler RC, Gordon LU (1995). Strategies for Measuring Stress in Psychiatric and Physical Disorders. Measuring Stress.

[72] Lazarus RS (1993). From psychological stress to the emotions: a history of changing outlooks. Annu Rev Psychol.

[73] Lazarus RS, Folkman S (1984). Stress, Appraisal, and Coping.

[74] Baum A, Blumenthal S, Matthews K, Weiss S (1993). Behavioral, Biological, and Environmental Interactions in Disease Processes. New Research Frontiers in Behavioral Medicine. Proceedings of the National Conference.

[75] Kirby ED, Williams VP, Hocking MC, Lane JD, Williams RB (2006). Psychosocial benefits of three formats of a standardized behavioral stress management program. Psychosom Med.

[76] Shiralkar MT, Harris TB, Eddins-Folensbee FF, Coverdale JH (2013). A systematic review of stress-management programs for medical students. Acad Psychiatry.

[77] Ma X, Yue ZQ, Gong ZQ, Zhang H, Duan NY, Shi YT, Wei GX, Li YF (2017). The effect of diaphragmatic breathing on attention, negative affect and stress in healthy adults. Front Psychol.

[78] Benson H, Klipper MZ (1975). The Relaxation Response.

[79] Kirk U, Gu X, Sharp C, Hula A, Fonagy P, Montague PR (2016). Mindfulness training increases cooperative decision making in economic exchanges: evidence from fMRI. Neuroimage.

[80] Kirk U, Gu X, Harvey AH, Fonagy P, Montague PR (2014). Mindfulness training modulates value signals in ventromedial prefrontal cortex through input from insular cortex. Neuroimage.

[81] Lutz A, Slagter HA, Dunne JD, Davidson RJ (2008). Attention regulation and monitoring in meditation. Trends Cogn Sci.

[82] Allen M, Dietz M, Blair KS, van Beek M, Rees G, Vestergaard-Poulsen P, Lutz A, Roepstorff A (2012). Cognitive-affective neural plasticity following active-controlled mindfulness intervention. J Neurosci.

[83] Zeidan F, Johnson SK, Diamond BJ, David Z, Goolkasian P (2010). Mindfulness meditation improves cognition: evidence of brief mental training. Conscious Cogn.

[84] Zeidan F, Emerson NM, Farris SR, Ray JN, Jung Y, McHaffie JG, Coghill RC (2015). Mindfulness meditation-based pain relief employs different neural mechanisms than placebo and sham mindfulness meditation-induced analgesia. J Neurosci.

[85] Zeidan F, Adler-Neal AL, Wells RE, Stagnaro E, May LM, Eisenach JC, McHaffie JG, Coghill RC (2016). Mindfulness-meditation-based pain relief is not mediated by endogenous opioids. J Neurosci.

[86] Quaglia JT, Zeidan F, Grossenbacher PG, Freeman SP, Braun SE, Martelli A, Goodman RJ, Brown KW (2019). Brief mindfulness training enhances cognitive control in socioemotional contexts: behavioral and neural evidence. PLoS One.

[87] White G, Taylor J (2011). The Natural History of Selborne.

[88] Shi J, Huang A, Jia Y, Yang X (2020). Perceived stress and social support influence anxiety symptoms of Chinese family caregivers of community-dwelling older adults: a cross-sectional study. Psychogeriatrics.

[89] Gong H, Ni CX, Liu YZ, Zhang Yi, Su WJ, Lian YJ, Peng W, Jiang CL (2016). Mindfulness meditation for insomnia: a meta-analysis of randomized controlled trials. J Psychosom Res.

[90] Thoma MV, Kirschbaum C, Wolf JM, Rohleder N (2012). Acute stress responses in salivary alpha-amylase predict increases of plasma norepinephrine. Biol Psychol.

[91] Ehlert U, Erni K, Hebisch G, Nater U (2006). Salivary alpha-amylase levels after yohimbine challenge in healthy men. J Clin Endocrinol Metab.

[92] Arnsten AFT (2009). Stress signalling pathways that impair prefrontal cortex structure and function. Nat Rev Neurosci.

[93] Pascoe MC, Thompson DR, Jenkins ZM, Ski CF (2017). Mindfulness mediates the physiological markers of stress: systematic review and meta-analysis. J Psychiatr Res.

[94] Sanada K, Montero-Marin J, Díez MA, Salas-Valero M, Pérez-Yus MC, Morillo H, Demarzo MMP, García-Toro M, García-Campayo J (2016). Effects of mindfulness-based interventions on salivary cortisol in healthy adults: a meta-analytical review. Front Physiol.

[95] Guan NC, Beng TS, Sue-Yin L, Kanagasundram S (2021). The effect of 5-min mindful breathing on pain in palliative care cancer patients: a randomized controlled study. Indian J Palliat Care.

[96] Ng CG, Lai KT, Tan SB, Sulaiman AH, Zainal NZ (2016). The effect of 5 minutes of mindful breathing to the perception of distress and physiological responses in palliative care cancer patients: a randomized controlled study. J Palliat Med.

