# Supplementary material for: App-Based Mindfulness for Attenuation of Subjective and Physiological Stress Reactivity in a Population With Elevated Stress: Randomized Controlled Trial
Source: JMIR Mhealth Uhealth. 2023 Oct 13;11:e47371. doi: 10.2196/47371 (PMC10612013; doi:10.2196/47371)
Supplement: Multimedia Appendix 1 [file mhealth_v11i1e47371_app1.docx]

**APPENDIX**

**Psychological measures**

**Perceived Stress Scale (PSS-10)**

The PSS consists of ten items designed to measure self-perceived stress [58]. Participants answer questions about their feelings and thoughts during the last month using a 5-point Likert scale (0 = never to 4 = very often). Scores range from 0–40 with higher scores indicating more stress. The scale has high test-retest reliability (α = .85).

**The Pittsburgh Sleep Quality Index (PSQI)**

The PSQI is a self-rated questionnaire which assesses sleep quality and disturbances over a one-month time interval [64]. Nineteen individual items generate seven component scores: subjective sleep quality, sleep latency, sleep duration, habitual sleep efficiency, sleep disturbances, use of sleeping medication, and daytime dysfunction. The sum of scores for these seven components yields one global score. The global score has a range of 0–21 points, where “0” indicates no sleep difficulty and “21” indicates severe sleep difficulties [64].

**Mindful Attention Awareness Scale (MAAS)**

The MAAS is a 15-item scale designed to assess trait or dispositional mindfulness [65]. Specifically, the MAAS measures attention to and awareness of the present moment. It has good psychometric qualities (Cronbach’s a=0.89). The 15 items are rated on a 6-point Likert scale. The MAAS has a range of 15-90 points and is scored by calculating the sum of the items and dividing by the total numbers of items. Higher scores indicate higher levels of trait mindfulness.

**Physiological measures**

**Acute stress manipulation**

The CPT is a standardized physiological acute stressor which has been shown to induce stress, reflected in autonomic stress reactivity [49,51,53,55,56]. The cold pressor used in the current study was a Lauda Alpha Heating and Cooling Thermostat. It measured the temperature in real time, while pumping water to uphold water flow and temperature throughout the cooler. If the participant was unable to keep their hand in the water, the experiment was terminated (n = 2; see *Participants*). During the CPT, the participants were asked to submerge their nondominant hand to the wrist in a 0-4 °C water bath for 3 minutes. To assess subjective levels of stress after the task, the participants rated how stressful they found the CPT on a scale of 1 (not stressful) to 10 (most stressful). The self-reported stress question was administered verbally immediately after completion of the CPT.

**Heart rate variability (HRV)**

**Physiological acquisition**

Heart rate (HR) was recorded as beat-to-beat intervals using the Firstbeat Bodyguard II HRV monitor (Firstbeat Technologies Ltd., Jyväskylä, Finland) that have been previously applied in research and validated with standard physiological monitoring systems used in clinical and laboratory settings [66,67]. The Bodyguard 2 is a wearable lightweight monitor attached to the chest using two ECG electrodes (Ambu Ltd., Ballerup, Denmark) for measuring HRV (RR-intervals). In the current study the HRV monitor was worn by participants during the acute stress manipulation task at pre and post (i.e. the CPT).

**Physiological signal processing**

The HRV measurements conducted in this study were performed according to the guidelines of the Task Force of the European Society of Cardiology and the North American Society of Pacing and Electrophysiology [59]. HRV collects data related to the change in the time intervals between consecutive heart beats and refers to an index of SNS activity and PNS activity at any given time [43]. Quantification of HRV parameters can broadly be classified into time and frequency domain measures. The primary time-domain measure is RMSSD and reflects the beat-to-beat variance in HR. RMSSD is typically used to estimate vagally mediated changes reflected in HRV [43]. A higher RMSSD generally suggests a more responsive and adaptable autonomic nervous system. Conversely, a lower RMSSD value may indicate reduced PNS activity and a less flexible autonomic system [45]. RMSSD is reported in milliseconds, with higher RMSSD indicating increased PNS activation [43,59]. In following these standardized procedures, we report RMSSD in the current study.

All raw physiological data was processed for time-domain parameters using the Kubios analysis software (version 3.4). The recorded data was imported to Kubios to calculate R-R intervals and associated variability [68]. Examination of the electrocardiogram data (ECG) ensured that the autonomic R-wave detection algorithm had been performed satisfactorily. Artifact removal for the HRV was performed manually using the artifact correction tool to detect R-R intervals provided by the Kubios software. When correction was applied, detected artifact beats were replaced using cubic spline interpolation. Because of the skewed distribution, the HRV variables were log transformed prior to exposing the data to statistical analysis. The HRV data was recorded continuously during the *rest period*, *CPT period*, and *recovery period* at both pre and post on a subject-by-subject basis. The HRV data was subsequently broken up into these three time-series.


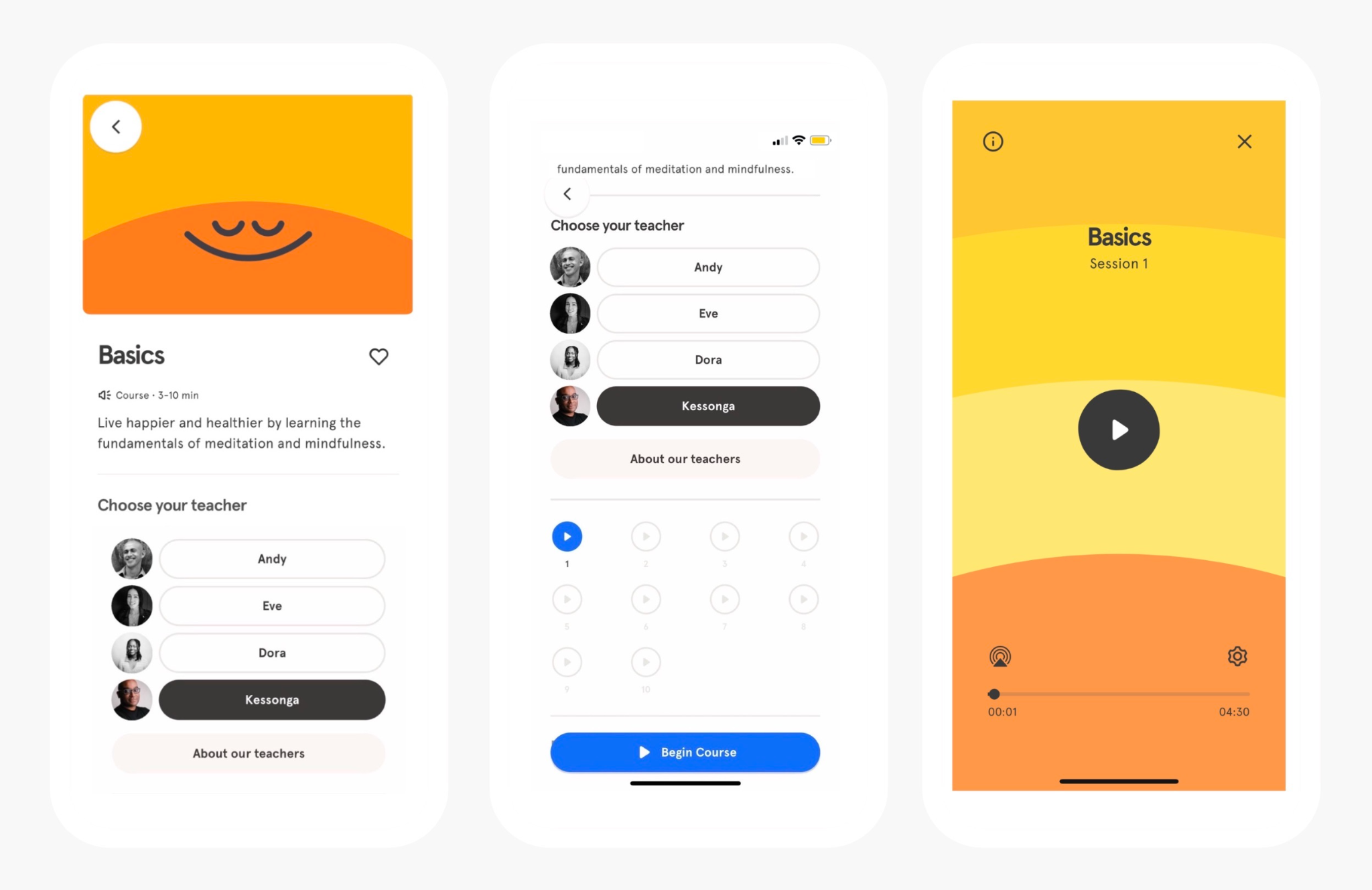


**Figure S1**. Basics (mindfulness) course


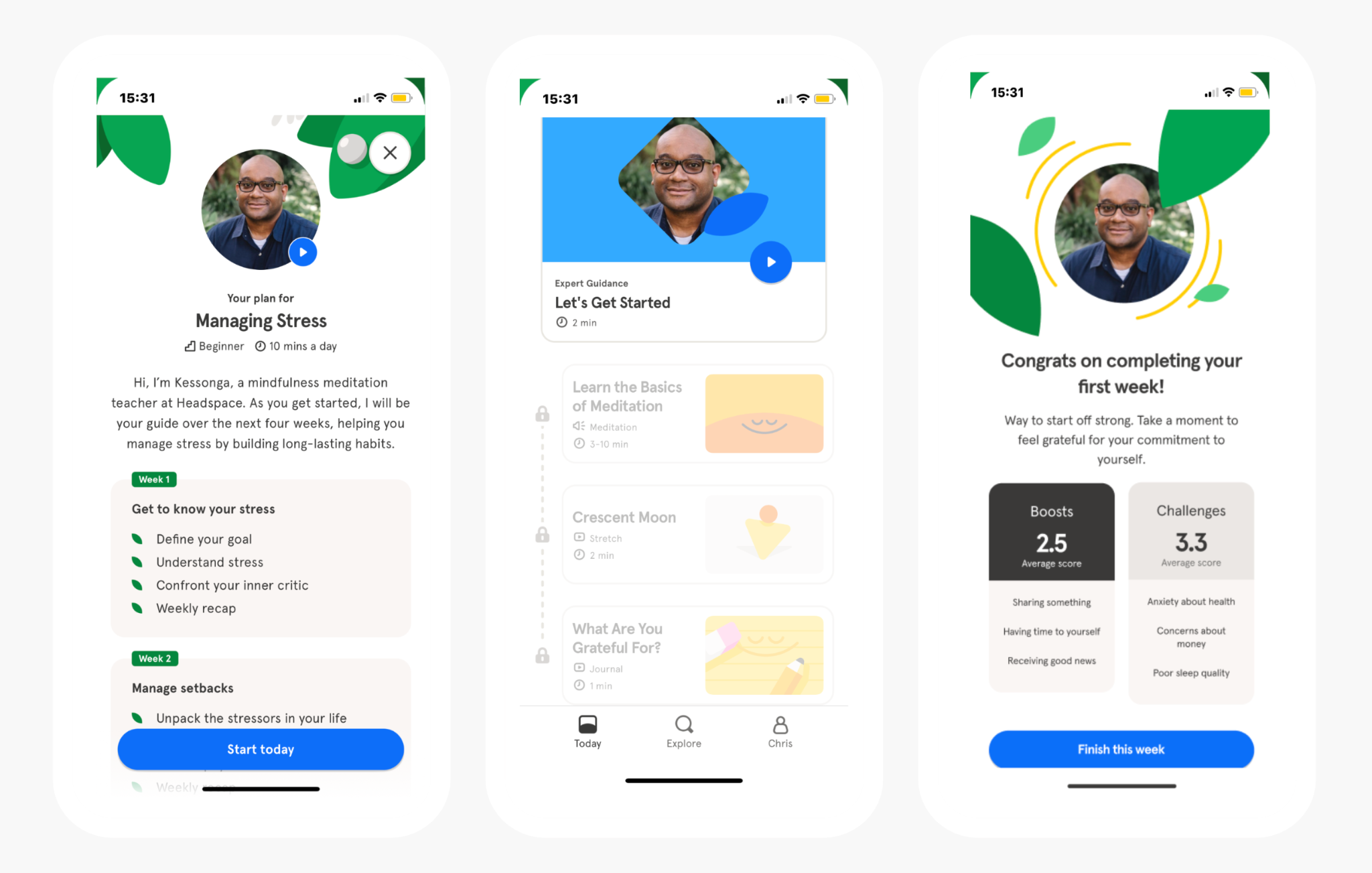


**Figure S2** Managing Stress (mindfulness) guided program.
