# Supplementary figures and images for: App-Based Mindfulness for Attenuation of Subjective and Physiological Stress Reactivity in a Population With Elevated Stress: Randomized Controlled Trial
Source: JMIR Mhealth Uhealth. 2023 Oct 13;11:e47371. doi: 10.2196/47371 (PMC10612013; doi:10.2196/47371)

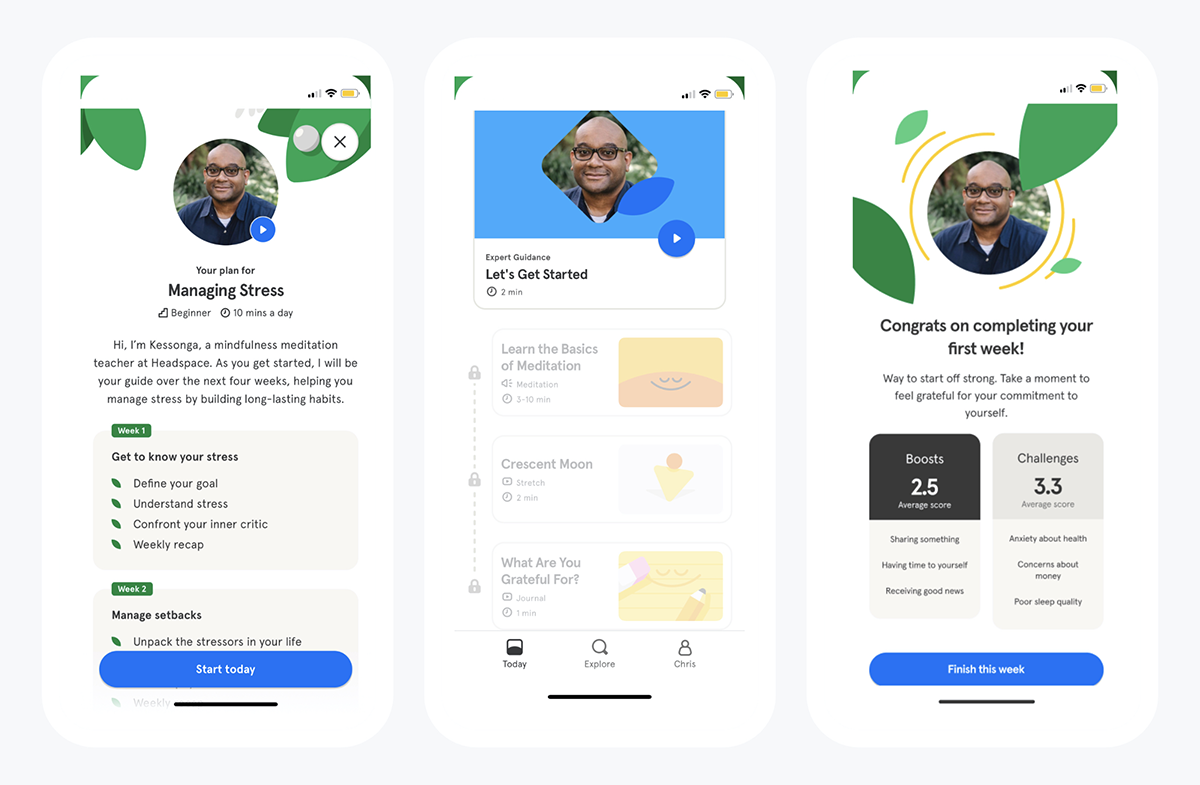

Supplement: Multimedia Appendix 2 [file mhealth_v11i1e47371_app2.png]

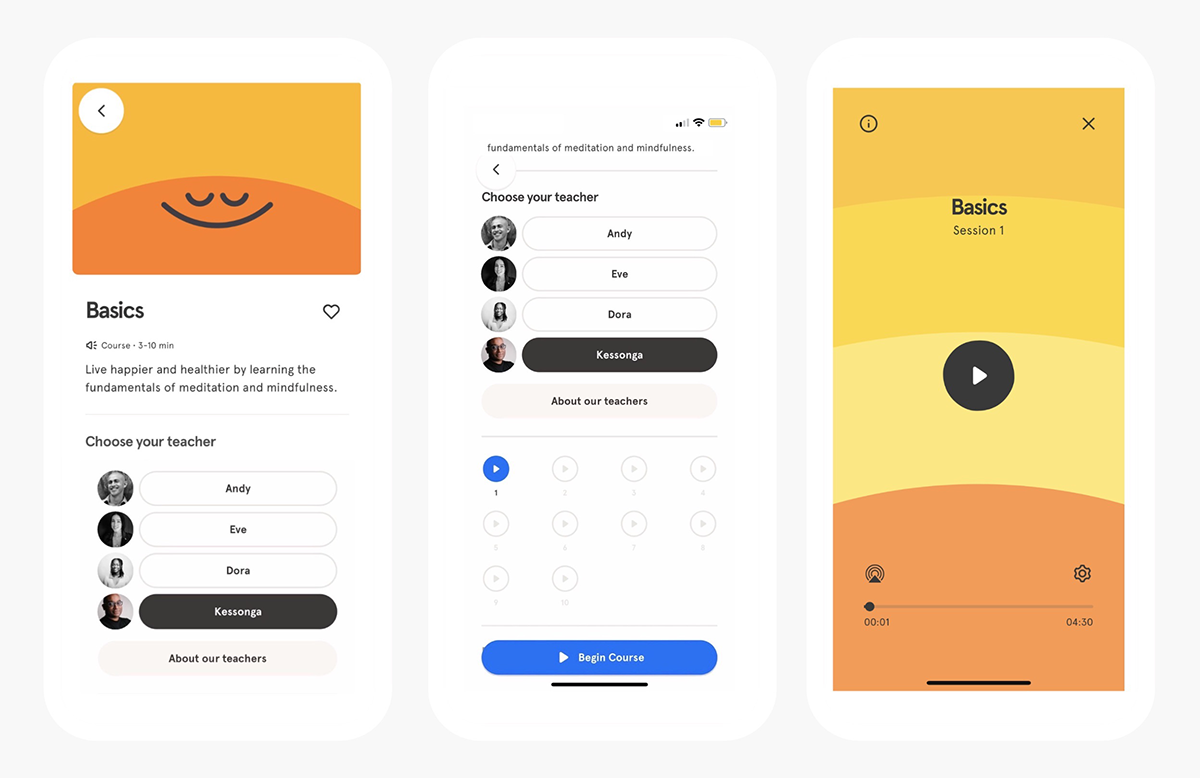

Supplement: Multimedia Appendix 3 [file mhealth_v11i1e47371_app3.png]
