# Supplementary material for: App-Based Mindfulness for Attenuation of Subjective and Physiological Stress Reactivity in a Population With Elevated Stress: Randomized Controlled Trial
Source: JMIR Mhealth Uhealth. 2023 Oct 13;11:e47371. doi: 10.2196/47371 (PMC10612013; doi:10.2196/47371)
Supplement: Multimedia Appendix 4 [file mhealth_v11i1e47371_app4.pdf]

CONSORT-EHEALTH Checklist V1.6.2 Report

	Manuscript Number	47371

(based on CONSORT-EHEALTH V1.6), available at [http://tinyurl.com/consort-ehealth-v1-6].

Date completed
9/7/2023 7:07:14
by
Ulrich Kirk

App-Based Mindfulness Passes the Stress Test: Attenuation of Subjective and Physiological Stress Reactivity in a Population with Elevated Stress

TITLE

1a-i) Identify the mode of delivery in the title
"App-Based Mindfulness"

1a-ii) Non-web-based components or important co-interventions in title
No room in title for mentioning specific intervention arms.

1a-iii) Primary condition or target group in the title
"Population with Elevated Stress"

ABSTRACT

1b-i) Key features/functionalities/components of the intervention and comparator in the METHODS section of the ABSTRACT
"Participants were randomly allocated to 1 of 5 groups: a digital guided program designed to alleviate stress (Managing Stress), a digital mindfulness fundamentals course (Basics), digitally delivered breathing exercises, an active control intervention (Audiobook), and a waitlist control group."

1b-ii) Level of human involvement in the METHODS section of the ABSTRACT
this was not included in abstract

1b-iii) Open vs. closed, web-based (self-assessment) vs. face-to-face assessments in the METHODS section of the ABSTRACT
"app-based guided mindfulness "

1b-iv) RESULTS section in abstract must contain use data
"The study included 163 participants who had moderate to high perceived stress as assessed by the Perceived Stress Scale (PSS-10)."

1b-v) CONCLUSIONS/DISCUSSION in abstract for negative trials
"These results demonstrate efficacy of app-based mindfulness in a population with moderate to high stress on improving self-reported stress, sleep quality, and physiological measures of stress during an acute stress manipulation task."

INTRODUCTION

2a-i) Problem and the type of system/solution
"This study examined if 3 different formats of digital mindfulness interventions demonstrated efficacy in terms of reducing self-reported levels of stress, sleep quality, and influencing physiological markers of stress reactivity in a population with elevated levels of stress."

2a-ii) Scientific background, rationale: What is known about the (type of) system
"several research studies suggest that the Headspace mindfulness app decreases subjective levels of stress [18-25] and increases sleep quality [64]. Despite growing demands and usage of digital mental health apps, there is a lack of evidence on the efficacy of these interventions in clinical populations [26,27]. "

Does your paper address CONSORT subitem 2b?
"We hypothesized (H1) that the app-based mindfulness interventions would yield significant improvements in self-reported stress (PSS-10) as compared to the active and waitlist control groups. We also hypothesized that trait mindfulness (MAAS) and sleep quality (PSQI) would improve in the mindfulness groups compared to control groups.
To probe physiological effects of stress, the study employed an acute stress manipulation task (ie, the CPT) at pre and post whilst measuring physiological activity (HRV) before, during, and after exposure to the acute stressor, and in addition self-reported stress perception immediately after stress exposure.
We hypothesized (H2) that the app-based mindfulness interventions would result in reduced physiological stress reactivity during exposure to the CPT, expressed as increased HRV activity compared to the active and waitlist control groups.
"

METHODS		
**3a) CONSORT: Description of trial design (such as parallel, factorial) including allocation ratio**		
"The study included 5 experimental groups using a prepost design."		
**3b) CONSORT: Important changes to methods after trial commencement (such as eligibility criteria), with reasons**		
there were no changes to methods after trial commencement		
**3b-i) Bug fixes, Downtimes, Content Changes**		
no important changes made on the intervention or comparator during the trial		
**4a) CONSORT: Eligibility criteria for participants**		
"A total of 225 research participants who had moderate to high perceived stress based on PSS-10 total scores (14-40) were recruited for the study"		
**4a-i) Computer / Internet literacy**		
Computer / Internet literacy was not assessed in the study		
**4a-ii) Open vs. closed, web-based vs. face-to-face assessments:**		
"The participants were recruited through flyers and advertisements at a local university (University of Southern Denmark) and the Region of Southern Denmark"		
**4a-iii) Information giving during recruitment**		
"Upon arrival for the first laboratory visit (ie, preintervention or T1), the participants initially signed the consent form. Participants were subsequently instructed on how to complete the experimental procedures (Figure 2)"		
**4b) CONSORT: Settings and locations where the data were collected**		
"The participants were recruited through flyers and advertisements at a local university (University of Southern Denmark) "		
**4b-i) Report if outcomes were (self-)assessed through online questionnaires**		
"The experimental procedures consisted of completing surveys (see psychological measures), and subsequently completing the CPT, that is, the stress manipulation whilst HRV was collected (see physiological measures). "		
**4b-ii) Report how institutional affiliations are displayed**		
**5) CONSORT: Describe the interventions for each group with sufficient details to allow replication, including how and when they were actually administered**		
**5-i) Mention names, credential, affiliations of the developers, sponsors, and owners**		
**5-ii) Describe the history/development process**		
**5-iii) Revisions and updating**		
**5-iv) Quality assurance methods**		
**5-v) Ensure replicability by publishing the source code, and/or providing screenshots/screen-capture video, and/or providing flowcharts of the algorithms used**		
**5-vi) Digital preservation**		
**5-vii) Access**		
"The interventions were completed using a custom-built research smartphone app. Participants were given access to a smartphone app that was built for the purpose of conducting scientific research at the University of Southern Denmark that contained the training content for each specific intervention. The app has been applied in previous scientific research studies [36,63,64]. The app was set up to contain specific content related to each of the interventions. That is, participants could only access the content in the app belonging to the specific type of intervention they were completing at any given time. "		
**5-viii) Mode of delivery, features/functionalities/components of the intervention and comparator, and the theoretical framework**		
"Participants did not receive an introductory session to the active intervention conditions but were provided with oral and written instructions about the app usage for the intervention period. Participants were instructed to follow the program in full."		
**5-ix) Describe use parameters**		

"Whereas the other 3 active interventions comprised 30 sessions, the Managing Stress intervention entailed 20 total sessions (5 sessions per week) with an average of 15 minutes duration per session. The Managing Stress intervention had the same total duration relative to the duration of the other 3 active interventions (approximately 300 min in total for each intervention), allowing us to investigate if the training frequency yielded a difference across the active interventions."		
**5-x) Clarify the level of human involvement**		
"Participants did not receive an introductory session to the active intervention conditions but were provided with oral and written instructions about the app usage for the intervention period. Participants were instructed to follow the program in full."		
**5-xi) Report any prompts/reminders used**		
there were no prompts or reminders used during the study		
**5-xii) Describe any co-interventions (incl. training/support)**		
all the intereventions used an app-based modality with identical time commitment. there were no support offered to participants during the study		
**6a) CONSORT: Completely defined pre-specified primary and secondary outcome measures, including how and when they were assessed**		
"Primary outcome measures were perceived stress using the PSS-10, self-reported sleep quality using the Pittsburgh Sleep Quality Index, and trait mindfulness using the Mindful Attention Awareness Scale"		
**6a-i) Online questionnaires: describe if they were validated for online use and apply CHERRIES items to describe how the questionnaires were designed/deployed**		
**6a-ii) Describe whether and how “use” (including intensity of use/dosage) was defined/measured/monitored**		
**6a-iii) Describe whether, how, and when qualitative feedback from participants was obtained**		
**6b) CONSORT: Any changes to trial outcomes after the trial commenced, with reasons**		
"The participants were recruited through flyers and advertisements at a local university (University of Southern Denmark) "		
**7a) CONSORT: How sample size was determined**		
**7a-i) Describe whether and how expected attrition was taken into account when calculating the sample size**		
**7b) CONSORT: When applicable, explanation of any interim analyses and stopping guidelines**		
"Primary outcome measures were perceived stress using the PSS-10, self-reported sleep quality using the Pittsburgh Sleep Quality Index, and trait mindfulness using the Mindful Attention Awareness Scale"		
**8a) CONSORT: Method used to generate the random allocation sequence**		
"The randomization procedure was determined after study recruitment, but before study launch. Specifically, participants were randomly allocated to 1 of 5 groups (Managing Stress, Basics, Breathing, Audiobook, or waitlist control) using sequence generation by the research team, who were not formally blinded to group allocation. "		
**8b) CONSORT: Type of randomisation; details of any restriction (such as blocking and block size)**		
"The randomization procedure was determined after study recruitment, but before study launch. Specifically, participants were randomly allocated to 1 of 5 groups (Managing Stress, Basics, Breathing, Audiobook, or waitlist control) using sequence generation by the research team, who were not formally blinded to group allocation. "		
**9) CONSORT: Mechanism used to implement the random allocation sequence (such as sequentially numbered containers), describing any steps taken to conceal the sequence until interventions were assigned**		
"Participants were not informed about group allocation until after completion of the preintervention experimental procedures"		
**10) CONSORT: Who generated the random allocation sequence, who enrolled participants, and who assigned participants to interventions**		
"The randomization procedure was determined after study recruitment, but before study launch. Specifically, participants were randomly allocated to 1 of 5 groups (Managing Stress, Basics, Breathing, Audiobook, or waitlist control) using sequence generation by the research team, who were not formally blinded to group allocation."		
**11a) CONSORT: Blinding - If done, who was blinded after assignment to interventions (for example, participants, care providers, those assessing outcomes) and how**		
**11a-i) Specify who was blinded, and who wasn’t**		

"The randomization procedure was determined after study recruitment, but before study launch. Specifically, participants were randomly allocated to 1 of 5 groups (Managing Stress, Basics, Breathing, Audiobook, or waitlist control) using sequence generation by the research team, who were not formally blinded to group allocation. Participants were not informed about group allocation until after completion of the preintervention experimental procedures"		
**11a-ii) Discuss e.g., whether participants knew which intervention was the "intervention of interest" and which one was the "comparator"**		
"The 3 types of mindfulness interventions were identical in total training dosage but varied in content and intervention length. That is, 2 of the 3 mindfulness interventions (Basics and Breathing) consisted of 30 sessions, whereas the third intervention (Managing Stress) consisted of 20 sessions. The type of content also varied whereby 2 interventions (Basics and Managing Stress) were programmatic that progressed from session to session, whereas the third interventions (Breathing) consisted of single succinct exercises. Furthermore, 1 intervention was designed specifically to reduce stress in people with elevated stress using mindfulness-based content (Managing Stress). This interventional setup allowed us to investigate if the unique characteristic of each intervention would result in differential efficacy in a population with moderate to high stress, while keeping the total training duration of each intervention identical. In other words, the rationale for employing 3 formats of mindfulness interventions was to explore if there were any differential effects between these distinct formats of mindfulness."		
**11b) CONSORT: If relevant, description of the similarity of interventions**		
"The 3 types of mindfulness interventions were identical in total training dosage but varied in content and intervention length. That is, 2 of the 3 mindfulness interventions (Basics and Breathing) consisted of 30 sessions, whereas the third intervention (Managing Stress) consisted of 20 sessions. The type of content also varied whereby 2 interventions (Basics and Managing Stress) were programmatic that progressed from session to session, whereas the third interventions (Breathing) consisted of single succinct exercises. Furthermore, 1 intervention was designed specifically to reduce stress in people with elevated stress using mindfulness-based content (Managing Stress). This interventional setup allowed us to investigate if the unique characteristic of each intervention would result in differential efficacy in a population with moderate to high stress, while keeping the total training duration of each intervention identical. In other words, the rationale for employing 3 formats of mindfulness interventions was to explore if there were any differential effects between these distinct formats of mindfulness."		
**12a) CONSORT: Statistical methods used to compare groups for primary and secondary outcomes**		
"A series of mixed groups (Managing Stress, Basics, Breathing, Audiobook, Waitlist control) × time (pretest, posttest) analysis of variance (ANOVAs) were performed on PSS-10, PSQI, MAAS, and CPT self-reported stress. "		
**12a-i) Imputation techniques to deal with attrition / missing values**		
"In total, 38 participants were excluded from the analysis either because they did not initiate the intervention practice (n=29), did not show up for the post-intervention laboratory visit (n=6) or were unable to complete the CPT procedure (n=3). 24 participants were excluded because they did not meet the minimum engagement dosage of 100 minutes (total engagement duration was 300 min). Thus, the total number of participants in our analysis was 163 participants. The rationale for the cut off at a minimum engagement dosage of 100 minutes was to ensure that there were no significant differences in engagement duration across the 4 active intervention groups (Table 1)."		
**12b) CONSORT: Methods for additional analyses, such as subgroup analyses and adjusted analyses**		
we did not include subgroup analyses in the current manscript		
RESULTS		
**13a) CONSORT: For each group, the numbers of participants who were randomly assigned, received intended treatment, and were analysed for the primary outcome**		
"In total, 38 participants were excluded from the analysis either because they did not initiate the intervention practice (n=29), did not show up for the post-intervention laboratory visit (n=6) or were unable to complete the CPT procedure (n=3). 24 participants were excluded because they did not meet the minimum engagement dosage of 100 minutes (total engagement duration was 300 min). Thus, the total number of participants in our analysis was 163 participants. The rationale for the cut off at a minimum engagement dosage of 100 minutes was to ensure that there were no significant differences in engagement duration across the 4 active intervention groups (Table 1)."		
**13b) CONSORT: For each group, losses and exclusions after randomisation, together with reasons**		
"In total, 38 participants were excluded from the analysis either because they did not initiate the intervention practice (n=29), did not show up for the post-intervention laboratory visit (n=6) or were unable to complete the CPT procedure (n=3). 24 participants were excluded because they did not meet the minimum engagement dosage of 100 minutes (total engagement duration was 300 min). Thus, the total number of participants in our analysis was 163 participants. The rationale for the cut off at a minimum engagement dosage of 100 minutes was to ensure that there were no significant differences in engagement duration across the 4 active intervention groups (Table 1)."		
**13b-i) Attrition diagram**		
since we excluded participants with less than 100 min training of a given intervention, we dont find that an attrition diagram would be relevant		
**14a) CONSORT: Dates defining the periods of recruitment and follow-up**		
the study did not include follow-up		

**14a-i) Indicate if critical “secular events” fell into the study period**		
**14b) CONSORT: Why the trial ended or was stopped (early)**		
the item is not relevant as the duration of the intervention was made explicit in the recruitment phase		
**15) CONSORT: A table showing baseline demographic and clinical characteristics for each group**		
see table 1 in the manuscript		
**15-i) Report demographics associated with digital divide issues**		
see table 1 in the manuscript, albeit we did not collect eg. ses in this study		
**16a) CONSORT: For each group, number of participants (denominator) included in each analysis and whether the analysis was by original assigned groups**		
**16-i) Report multiple “denominators” and provide definitions**		
the item is not relevant as the the analysis was by original assigned groups		
**16-ii) Primary analysis should be intent-to-treat**		
**17a) CONSORT: For each primary and secondary outcome, results for each group, and the estimated effect size and its precision (such as 95% confidence interval)**		
included where relevant in the manuscript		
**17a-i) Presentation of process outcomes such as metrics of use and intensity of use**		
**17b) CONSORT: For binary outcomes, presentation of both absolute and relative effect sizes is recommended**		
included where relevant in manuscript		
**18) CONSORT: Results of any other analyses performed, including subgroup analyses and adjusted analyses, distinguishing pre-specified from exploratory**		
item is not applicable/relevant for the study as no subgroup analyses were included		
**18-i) Subgroup analysis of comparing only users**		
**19) CONSORT: All important harms or unintended effects in each group**		
item is not applicable as there were no unintended effects in each group		
**19-i) Include privacy breaches, technical problems**		
**19-ii) Include qualitative feedback from participants or observations from staff/researchers**		
DISCUSSION		
**20) CONSORT: Trial limitations, addressing sources of potential bias, imprecision, multiplicity of analyses**		
**20-i) Typical limitations in ehealth trials**		
"Strengths and Limitations<br>Although the results of this study are promising regarding the efficacy in 2 of the 3 app-based mindfulness interventions, several limitations must be noted. First, the sample primarily comprised young university students in their twenties, thus the generalizability of our findings may be limited. Second, this study did not investigate the efficacy of the app-based interventions beyond 30 days, nor whether any of the findings were maintained (regardless of app usage) beyond this period. Future studies should consider including follow-up measurements to evaluate sustained outcomes. Third, the study did not collect endocrine measures such as cortisol to assess the acute stress response during the CPT, although the HRV-data supported, it would have been interesting to also inspect correlations between endocrine measures and HRV to be completely certain that the CPT elicited reliable stress reactivity in participants. Fourth, participants were instructed to follow the program in full, however training adherence was not checked during the intervention, and participants were not reminded by the researchers to complete the daily training in the intervention period, which might have increased adherence.<br>"		
**21) CONSORT: Generalisability (external validity, applicability) of the trial findings**		
**21-i) Generalizability to other populations**		

**21-ii) Discuss if there were elements in the RCT that would be different in a routine application setting**		
**22) CONSORT: Interpretation consistent with results, balancing benefits and harms, and considering other relevant evidence**		
**22-i) Restate study questions and summarize the answers suggested by the data, starting with primary outcomes and process outcomes (use)**		
"Principal Findings<br>This study sought to investigate if 3 formats of digital mindfulness interventions would show efficacy in reducing subjective and physiological levels of stress in a population with elevated symptoms of stress. The study found that self-reported stress as measured by PSS-10 was significantly reduced in the Managing Stress and Basics groups from pre to post compared to the other groups. Furthermore, the study found significant improvement in self-reported sleep quality as measured by the PSQI in the Managing Stress and Basics groups from pre to post compared to the other groups. Trait mindfulness, as measured by MAAS, did not yield significant differences across any of the groups. Finally, the results showed that only the Basics and Managing Stress groups displayed significantly reduced levels of physiological stress (ie, expressed as increased HRV activity) during exposure to the acute stress manipulation task (ie, CPT).<br>"		
**22-ii) Highlight unanswered new questions, suggest future research**		
Other information		
**23) CONSORT: Registration number and name of trial registry**		
Registration: ClinicalTrials.gov NCT05832632; https://www.clinicaltrials.gov/ct2/show/NCT05832632		
**24) CONSORT: Where the full trial protocol can be accessed, if available**		
Registration: ClinicalTrials.gov NCT05832632; https://www.clinicaltrials.gov/ct2/show/NCT05832632		
**25) CONSORT: Sources of funding and other support (such as supply of drugs), role of funders**		
"This study was funded by Headspace."		
**X26-i) Comment on ethics committee approval**		
**x26-ii) Outline informed consent procedures**		
**X26-iii) Safety and security procedures**		
**X27-i) State the relation of the study team towards the system being evaluated**		
